# Genetic and Physiological Effects of Insulin on Human Urate Homeostasis

**DOI:** 10.3389/fphys.2021.713710

**Published:** 2021-08-02

**Authors:** Asim K. Mandal, Megan P. Leask, Christopher Estiverne, Hyon K. Choi, Tony R. Merriman, David B. Mount

**Affiliations:** ^1^Renal Division, Brigham and Women’s Hospital, Harvard Medical School, Boston, MA, United States; ^2^Biochemistry Department, University of Otago, Dunedin, New Zealand; ^3^Division of Rheumatology and Clinical Immunology, University of Alabama, Birmingham, AL, United States; ^4^Division of Rheumatology, Massachusetts General Hospital, Harvard Medical School, Boston, MA, United States; ^5^Renal Division, VA Boston Healthcare System, Harvard Medical School, Boston, MA, United States

**Keywords:** insulin receptor (INSR), serum urate (SU), proximal tubule epithelial cell line (PTC-05), glucose transporter-9 (GLUT9), a urate transporter(GLUT9), organic anion transporter (OAT), insulin receptor substrate (IRS1), phosphoinositide 3-kinase (PI3K)

## Abstract

Insulin and hyperinsulinemia reduce renal fractional excretion of urate (FeU) and play a key role in the genesis of hyperuricemia and gout, via uncharacterized mechanisms. To explore this association further we studied the effects of genetic variation in insulin-associated pathways on serum urate (SU) levels and the physiological effects of insulin on urate transporters. We found that urate-associated variants in the human insulin (INS), insulin receptor (INSR), and insulin receptor substrate-1 (IRS1) loci associate with the expression of the insulin-like growth factor 2, IRS1, INSR, and ZNF358 genes; additionally, we found genetic interaction between *SLC2A9* and the three loci, most evident in women. We also found that insulin stimulates the expression of GLUT9 and increases [^14^C]-urate uptake in human proximal tubular cells (PTC-05) and HEK293T cells, transport activity that was effectively abrogated by uricosurics or inhibitors of protein tyrosine kinase (PTK), PI3 kinase, MEK/ERK, or p38 MAPK. Heterologous expression of individual urate transporters in *Xenopus* oocytes revealed that the [^14^C]-urate transport activities of GLUT9a, GLUT9b, OAT10, OAT3, OAT1, NPT1 and ABCG2 are directly activated by insulin signaling, through PI3 kinase (PI3K)/Akt, MEK/ERK and/or p38 MAPK. Given that the high-capacity urate transporter GLUT9a is the exclusive basolateral exit pathway for reabsorbed urate from the renal proximal tubule into the blood, that insulin stimulates both GLUT9 expression and urate transport activity more than other urate transporters, and that *SLC2A9* shows genetic interaction with urate-associated insulin-signaling loci, we postulate that the anti-uricosuric effect of insulin is primarily due to the enhanced expression and activation of GLUT9.

## Introduction

Hyperuricemia and gout have a strong association with metabolic syndrome, insulin resistance, and type 2 diabetes (Choi and Ford, 2007; Choi et al., 2007, 2008). Multiple studies have thus demonstrated a positive relationship between serum insulin and elevated serum urate (SU) levels, in healthy volunteers and people with diabetes (Modan et al., 1987; Facchini et al., 1991; Vuorinen-Markkola and Yki-Jarvinen, 1994; Quinones Galvan et al., 1995; Muscelli et al., 1996; Kodama et al., 2009; Robles-Cervantes et al., 2011; Gill et al., 2013; MacFarlane et al., 2015; Perez-Ruiz et al., 2015). Notably, hyperuricemic mice develop insulin resistance and suffer from impaired glucose tolerance (Zhu et al., 2014) suggesting possible inhibition of insulin signaling in hyperuricemia. However, Mendelian randomization studies show that urate homeostasis does not affect the development of type 2 diabetes (Johnson et al., 2018) or hyperinsulinemia (McCormick et al., 2021), suggesting that in humans hyperinsulinemia leads to urate retention and hyperuricemia rather than the converse. Indeed, physiological euglycemic hyperinsulinemia induced by insulin infusion (6 pmol/min/kg) in healthy volunteers acutely reduces urinary urate (25–35%) (Quinones Galvan et al., 1995; Muscelli et al., 1996; Ter Maaten et al., 1997), suggesting a key role for insulin in the pathogenesis of hyperuricemia. The kidney has thus been proposed as an “unwilling accomplice” in the sodium and urate retention associated with the metabolic syndrome, given preserved renal insulin sensitivity in the face of systemic insulin resistance (Reaven, 1997). At present, however, the underlying mechanisms for insulin-associated urate retention are only partially characterized. In rats, the administration of insulin decreased urinary urate excretion, with concurrent increased expression of a major urate reabsorption transporter, URAT1, and decreased expression of a major urate secretory transporter, ABCG2 (Toyoki et al., 2017). Exposure to insulin also increased the expression of endogenous URAT1 in NRK-52E cells and kidney epithelial cells (Toyoki et al., 2017). There is increased expression of GLUT9 in the placenta of insulin-dependent women (Stanirowski et al., 2017) and in the kidneys and liver of streptozotocin-induced diabetic mice (Keembiyehetty et al., 2006). In streptozotocin-induced diabetic rats, insulin administration reduced increased urinary excretion of urate and Na^+^ (Toyoki et al., 2017). In cultured cortical neurons subjected to oxidative stress, insulin increases intracellular urate concentration (Duarte et al., 2005). Notably, of crucial importance, results from animal models are often poorly reflective of human urate physiology, given the major genetic and physiological differences in the relevant pathways in humans versus rodents and other non-primate mammals (Mandal and Mount, 2015).

**FIGURE 1:**
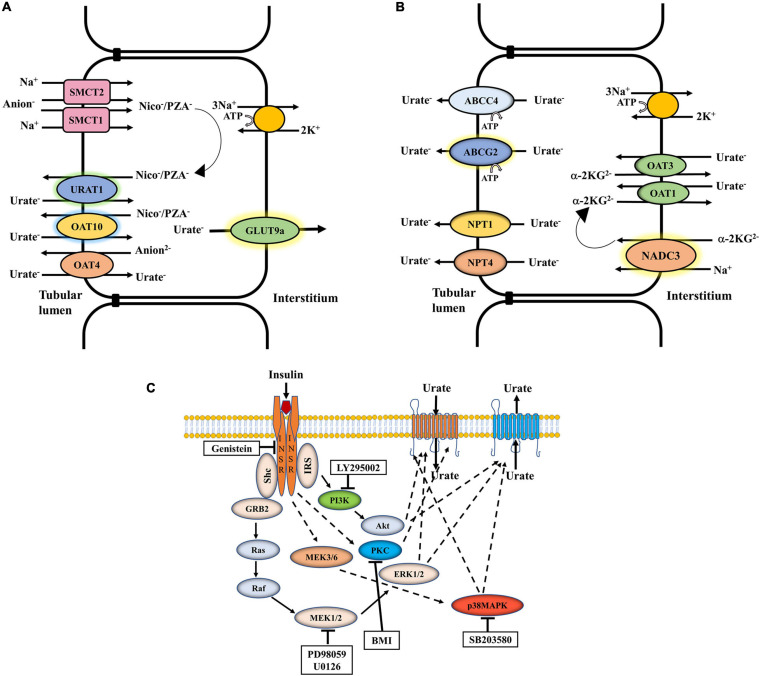
The net excretion of urate in the human urine depends on the balance between urate reabsorption and secretion, mediated by separate sets of urate transporters in the proximal tubule. **(A)** Urate reabsorption: The sodium-driven SMCT1 and SMCT2 transporters increase the intracellular pool of organic anions such as nicotinate or pyrazinoate (PZA), which function in urate/anion exchange mediated by URAT1 and OAT10. OAT4 in contrast exchanges apical urate for divalent anions. The membrane potential-driven GLUT9a functions as the exclusive exit pathway for reabsorbed urate from proximal tubule into blood. **(B)** Urate secretion: In a sodium dependent process involving the collaboration of NADC3, urate enters at the basolateral membrane in exchange with alpha-ketoglutarate, mediated by OAT1 and OAT3. Urate is transported across the apical surface into the tubular lumen via MRP4, ABCG2, NPT1, and NPT4. **(C)** Regulation of urate transport by insulin signaling: Insulin binding to its receptor (INSR) leads to the activation of insulin receptor substrate (IRS), Akt, ERK1/2, p38 MAPK and PKC. The general effect of insulin on urate transport (reabsorption or secretion) is indicated. Genistein, a phosphotyrosine kinase (PTK)-specific inhibitor; LY 295002, a PI3K-specific inhibitor; PD98059, a MEK/ERK-specific inhibitor; SB 203580, a p38 MAPK-specific inhibitor; BMI, bisindolylmaleimide 1-hydrochloride, a protein kinase C (PKC)-specific inhibitor.

The net excretion of urate in the urine reflects the balance between urate re-absorption and secretion within the proximal tubule, each process mediated by a separate set of urate transporters (Figures 1A,B). The urate transporters URAT1 and OAT10, co-expressed in the apical membrane of renal proximal tubule cells (Bahn et al., 2008), reabsorb urate in exchange with intracellular nicotinate, pyrazinoate (PZA), or related monocarboxylates (Enomoto et al., 2002; Mandal et al., 2017)**;** the sodium monocarboxylate transporters SMCT1 and SMCT2 facilitate intracellular accumulation of these anions, resulting in “trans-activation” of apical exchange with urate (Figure 1A; Mandal and Mount, 2015; Mandal et al., 2017). Dysfunctional variants of URAT1 (Enomoto et al., 2002) and OAT10 (Higashino et al., 2020) are associated with decreased SU levels, underscoring the importance of these apical transporters in urate re-absorption. GLUT9 (expressed as two isoforms, GLUT9a and GLUT9b) is a membrane-potential driven, high-capacity urate transporter (Anzai et al., 2008; Caulfield et al., 2008; Vitart et al., 2008; Witkowska et al., 2012; Mandal et al., 2017), functioning as the sole transporter for exclusive exit of reabsorbed urate from proximal tubule into blood. Notaby, variation in *SLC2A9*, the gene that encodes GLUT9, has the biggest single-gene effect on SU levels of all the multiple genes that influence urate homeostasis (Doring et al., 2008). In the urate secretion pathway, the organic anion exchangers OAT1 and OAT3, expressed at the basolateral membrane of the proximal tubule, export urate from blood (Sweet et al., 2003; Mandal and Mount, 2015) with subsequent secretion at the apical membrane via electrogenic urate transporters, NPT1 and NPT4 (Jutabha et al., 2003; Iharada et al., 2010) and ATP-driven urate transporters, ABCG2 (Figure 1B; Woodward et al., 2009; Matsuo et al., 2011; Mandal et al., 2017) and ABCC4 (Tanner et al., 2017). Notably, single cell RNA sequencing data (RNA-seq) from human kidney (Wu et al., 2018a,b) indicates that these re-absorptive and secretory pathways are collectively co-expressed along the entire proximal tubule, rather than in separate cell types as proposed in the historical four-component model of renal urate transport; see Mandal and Mount (2015) for a critical review of the flawed four-component model.

Insulin is a key regulator of energy metabolism. It exerts its action through binding and activating its cell-surface receptor (INSR), a hetero-tetrameric transmembrane glycoprotein (disulfide linked into an α2β2 complex) belonging to the large class of the tyrosine kinase receptor family. Insulin signaling is conserved across species (Barbieri et al., 2003). Insulin binding to the α-subunits of the INSR activates the intracellular tyrosine kinase domain of the β-subunit that activates the insulin receptor substrate (IRS-1, IRS-2) protein complex through tyrosine phosphorylation (Shepherd et al., 1998), which in turn triggers phosphoinositide 3-kinase (PI3K) activity (Saltiel and Kahn, 2001) and induces phosphorylation-dependent activation of downstream signaling molecules, including Akt/protein kinase B (Akt/PKB), MEK/ERK (Kayali et al., 2000; Harmon et al., 2004), p38 MAPK (Niu et al., 2003) and atypical protein kinase C ζ (PKCζ) (Figure 1C; Shepherd et al., 1998; Saltiel and Kahn, 2001; Huang et al., 2004). Polymorphisms in the *INSR* and *IRS-1* genes associate with type 2 diabetes mellitus (T2DM) and insulin resistance (Almind et al., 1996; Rung et al., 2009). There is some evidence for insulin-signaling in the regulation of urate homeostasis; the neuroprotection of dopaminergic neurons by urate is thus abolished in presence of PI3K inhibitors (Gong et al., 2012) indicating a regulatory role for PI3K in neuronal urate transport. The activation of Akt, ERK and/or PKCζ stimulates the function of OAT3 and OAT1 (Soodvilai et al., 2005; Barros et al., 2009). However, again, there has been no systematic appraisal of the effects of insulin-related signaling on urate transporters.

## Materials and Methods

### Genetic Analysis

Summary level GWAS data for serum urate as outcome were sourced from Köttgen et al. (2013) and Tin et al. (2019). Expression quantitative trait locus (eQTL) data were obtained from the Genotype and Tissue Expression (GTEx, v8) database. Testing for co-localization of GWAS and eQTL signals in *cis* using COLOC2 was done based on the method previously described. COLOC2 is a Bayesian method that compares four different statistical models at a locus: no causal variant in the GWAS or eQTL region; a causal variant in either region, but not both; different causal variants in each region; or a shared causal variant in each region. Genes that had a posterior probability of co-localization greater than 0.8 were considered to have a shared causal variant.

Analysis of epistasis between pairs of SNPs was done in the United Kingdom Biobank dataset (project 12611) in which an interaction term was added to linear regression association analysis of genetic variants against serum urate level. The dataset analyzed was 472,684 individuals of European ancestry, of whom 54.5% were women. The average age was 56.8 years.

### Animals, Cell Lines and Reagents

Mature female *Xenopus laevis* frogs were purchased from NASCO (Fort Atkinson, WI, United States). A human kidney proximal tubule epithelial cell line (PTC-05) (Orosz et al., 2004) was obtained from Ulrich Hopfer (Case Western Reserve University, Cleveland, OH, United States). Human embryonic kidney cell line (HEK 293T) and other cell line**s** were obtained from ATCC (Manassas, VA, United States). DMEM and HAM’S F12 media, fetal bovine serum (FBS), insulin, human epidermal growth factor (EGF), were purchased from Invitrogen (Carlsbad, CA, United States). Type IV collagen, transferrin, dexamethasone, interferon-gamma, insulin powder/crystals, ascorbic acid, sodium selenite (Na_2_SeO_3_), and triiodothyronine (T3) were purchased from SIGMA (St Louis, MO, United States). Affinity purified rabbit anti-SLC2A9/GLUT9, anti-SLC22A12/URAT1 and anti-SLC22A13/ORCTL3/OAT10 antibodies were purchased from MBL (Medical and Biological Laboratories Co., Ltd., Woburn, MA, United States). Rabbit anti-insulin receptor beta (4 B8) antibody, anti-phospho IRS1 (Ser318), anti-IRS1, anti-phospho-Akt (Ser473), anti-Akt, anti-phospho-p44/42 MAPK(ERK1/2)(Thr202/Tyr204), anti-p44/42 MAPK(ERK1/2), anti-phospho-p38 MAPK (Thr180/Tyr182), anti-p38 MAPK antibodies, anti-ABCG2, and anti-β-actin, were purchased from Cell Signaling Technology (Danvers, MA, United States). Rabbit anti-NPT1 antibody was purchased from Thermo Fisher Scientific (Waltham, MA, United States). HRP-conjugated anti-rabbit IgG secondary antibody was purchased from BIO-RAD (Hercules, CA, United States). ECL solution was purchase from Thermo Scientific (Rockford, IL, United States). Genistein (soybean), LY 294002, bisindolylmaleimide 1-hydrochloride (BM1), PD 98059, SB203580, and U0126 were purchased from Calbiochem (Bilerica, MA, United States). The [^14^C]-urate (specific activity: 50 mCi/mmol) was purchased from Moravec Inc. (Brea, CA, United States).

### Cell Culture

All cells were routinely maintained in their respective appropriate growth medium in a humidified incubator at 37°C with 5% CO_2_. Human PTC-05 cells were grown as described previously (Orosz et al., 2004) on a type IV collagen-coated petri dish. HEK 293T cells and other cells as indicated were grown in Dulbecco’s modified Eagle’s medium (DMEM) following supplier’s instructions.

### Expression Constructs

For expression of human urate transporters (GLUT9a, GLUT9b, URAT1, OAT10, OAT1, OAT3, OAT4, NPT1, and ABCG2) in oocytes, the full-length coding region of complementary DNAs (cDNAs) were cloned into the 3.022 kb pGEMHE vector, which optimizes cRNA for expression in *Xenopus laevis* oocytes.

### Site-Directed Mutagenesis

Mutations in GLUT9 were introduced by site-directed mutagenesis PCR reaction using the QuickChange II site-directed mutagenesis kit (Agilent Technologies, Santa Clara, CA, United States) following the manufacturer’s instructions. Primer sequences for introducing mutations into the N-terminal domains or designing constructs with deletion of N-terminal domains of GLUT9 isoforms are listed in Supplementary Materials.

### Functional Expression in *Xenopus* Oocytes

Studies using *Xenopus laevis* oocytes have been carried out in accordance with the Guide for the Care and Use of Laboratory Animals as adopted and promulgated by the United States National Institutes of Health, and were approved by the Institution’s Animal Care and use Committee. Mature female *Xenopus laevis* frogs were subjected to partial ovariectomy under tricane anesthesia (0.17**%** for 15–20 min) followed by defolliculation of the oocytes as described previously (Mandal et al., 2017). The various *Xenopus laevis* expression constructs in pGEMHE were linearized by Not1, Nhe1 or EcoR1 digestion. The cRNAs were *in vitro* synthesized by using T7 RNA polymerase (mMESSAGE mMACHINE; Ambion, Austin, TX, United States) following the supplier’s protocol, isopropanol-precipitated, washed twice with 70% ethanol, dried, dissolved in sterile nuclease-free water and then stored at −80°C. The yield and integrity of the capped cRNA samples were assessed by spectroscopy (at 260 nm) and 1% agarose-formaldehyde gel electrophoresis, respectively. About 18 h after isolation, oocytes were microinjected with 50 nl of sterile water, or 50 nl of a cRNA solution containing 25/12.5 ng of the indicated cRNA using fine-tipped micropipettes by a microinjector (World Precision Instrument Inc., Sarasota, FL, United States) and then incubated in ND96 medium supplemented with pyruvate for 24 h for protein expression.

### RNA Extraction and RT-PCR

Total RNA from PTC-05 and other cells as indicated was extracted using spin columns with the RNeasy Mini Kit (QIAGEN, GmbH, Germany) following the manufacturer’s instructions. About 2 μg of total RNA, isolated from cells, were primed with poly-dT and random hexamers and then reverse-transcribed using AMV reverse transcriptase (New England Biolabs, Ipswich, MA, United States). Equal amount of cDNA was used for PCR amplification keeping a negative control lacking template cDNA. Primers utilized for RT-PCR are listed in Supplementary Materials. All PCR products were confirmed by cloning and sequencing.

### Uptake and Efflux Assays

The [^14^C]-urate uptake and efflux experiments in *Xenopus* oocytes were performed as described previously (Mandal et al., 2017; Mandal and Mount, 2019). To examine the effect of insulin on [^14^C]-urate uptake mediated by endogenous functional urate transporters in PTC-05 and HEK 293T cells, equal numbers (3×10^6^) of PTC-05 or HEK 293T cells were incubated with insulin (0.1 μM to 1.5 μM) in potassium-free (Cicirelli et al., 1988, 1990) and serum-free defined isotonic medium (143 mM NaCl, 1.8 mM CaCl_2_, 1 mM MgCl_2_, 5 mM HEPES, pH 7.4) for 30 min at 25°C and then subjected to [^14^C]-urate uptake in the same medium for 1 h at 25°C. For uptake experiments each group has three separate wells of cells (typically 3 × 10^6^/well), urate uptakes are expressed as pmol/3 × 10^6^ cells/h, results are reported as means ± S. E., and statistical significance is defined as two-tailed *p* < 0.05; each experiment is repeated three times. The [^14^C]-urate uptake activity was measured in isotonic K^+^-free uptake medium containing 20 μM [^14^C]-urate in 12-well plates after 1h of incubation at ∼25°C. All uptake experiments using oocytes included at least 20 oocytes in each experimental group, as described (Mandal et al., 2017; Mandal and Mount, 2019), using 40 μM [^14^C]-urate. For [^14^C]-urate efflux studies, control oocytes or oocytes expressing ABCG2 were preinjected with 50 nl of 1500 μM [^14^C]-urate dissolved in efflux medium (K^+^-free medium, pH 7.4). Statistical significance was defined as two-tailed *p* < 0.05, and results were reported as means ± S. E. All the uptake experiments shown were performed more than three times for confirmation; data shown for each figure are from a single representative experiment.

### Western Blotting

Western blotting was performed as described previously (Mandal et al., 2017). About 48 h post- microinjection, oocytes (∼100) were lysed using a Teflon homogenizer in lysis buffer (50 mM Tris–HCl, pH 7.5, 50 mM NaCl, 1 mM EDTA, pH 8, 1% Triton X-100) supplemented with protease inhibitors cocktail (Roche, Indianapolis, IN, United States). Western blotting was performed using appropriate primary and secondary antibodies. About 30 μg of total protein of lysates was loaded per lane and fractionated using 8% SDS/PAGE gel electrophoresis and then transferred to polyvinylidene difluoride (PVDF) membrane. All the Western blotting experiments were performed more than three times for confirmation; data shown for each figure are from a single representative experiment. Quantitative analysis of the intensity of protein bands in Western blots was performed using KwikQuant Image Manager software (Kindle Biosciences, Greenwich, CT, United States).

### Statistics

Statistical analyses including linear regressions and significance were determined by Student’s t test using SigmaPlot software. Transformation of data and curve fitting were made with SigmaPlot (Systat Software, Bangalore, Karnataka, India).

## Results

### Genetic Association of the *INS*, *INSR* and *IRS-1* Loci With Serum Urate Levels

Using publicly available SU genome-wide association data (Tin et al., 2019), signals of association (*p* < 5 × 10^–6^) were observed at *INS (β = −0.03 mg/dL, p = 1.50 × 10^–8^), INSR (β = 0.04, p = 1.07 × 10^–20^)* and *IRS1 (β = 0.02 mg/dL, p = 5.31 × 10^–6^)* (Supplementary Figure 1). To test if the signals of genetic association control SU levels through effects on expression of the *INS, INSR or IRS-1* genes, we used the Gene and Tissue Expression (GTEx, v8) database to test for co-localization of the serum urate genetic signals with genetic control of gene expression. This analysis revealed no colocalization of signal with expression of any genes in cis at the *INS* locus. However, we note that the equivalent analysis of Tin et al. (2019) using GTEx v6 reported colocalization [posterior probability of colocalization (PPC) > 0.8] of the signal with *IGF2* expression in brain and testis. The urate-raising allele at *IGF2* (rs35506085) decreases expression. At *INSR* there are two genetically independent GWAS signals. The maximal urate signal from Tin et al., 2019 marked by rs10405423 (Tin et al., 2019) co-localizes with *ZNF358* expression in adipose tissue (PPC = 0.90) with decreased expression of ZNF358. Whereas INSR expression did not colocalize (PPC = 0.03) with the urate genetic association data from Tin et al. (2019). The PPC in this case is likely confounded by the stronger rs10405423 primary association in the genetic association data. However, using the genetic association data from the Köttgen et al. (2013) GWAS Köttgen et al., 2013, *INSR* (lung) expression colocalises with urate genetic association (PPC = 0.96). The urate-raising GWAS allele of rs1035942 increases expression of *INSR*. At the IRS1 locus, the urate genetic association signal co-localizes with an eQTL (expression quantitative trait locus) for IRS-1 in the thyroid (PPC = 0.74). The urate-raising allele of rs61009233 associates with decreased expression of IRS1.

### Genetic Interaction (Epistasis) Between *SLC2A9* and *IRS1 / IGF2 / ZNF358*

Using the United Kingdom Biobank dataset, the lead urate-associated SNP (Tin et al., 2019) at *SLC2A9* (rs4447862) was tested for epistatic interaction with the lead SNP at each of *IRS1*, *IGF2* and *INSR* (*rs61009233*, *rs35506085*, and *rs10405423*, respectively) by adding an interaction term into a multivariable linear regression model including *rs4447862* and the second SNP as independent variables. Given the stronger genetic effect of *SLC2A9* on serum urate levels in women (Köttgen et al., 2013), the analyses were done stratified by sex, adjusting by age. There was evidence for epistatic interaction for *SLC2A9* with *IRS1*, *IGF2* and *ZNF358* in men (β = −1.271 μmol/L, SE = 0.641, β = 0.106 μmol/L, SE = 0.646, β = −2.025 μmol/L, SE = 0.781) and women (β = −2.682 μmol/L, SE = 0.515, β = −1.333 μmol/L, SE = 0.520, β = −2.534 μmol/L, SE = 0.627). There was no evidence for heterogeneity in interaction between men and women (all *p* > 0.05). The nature of the interactions was investigated by standard linear regression of the 9 genotype groups (Table 1). For each of the 3 SNP groupings the interaction was driven by a non-additive reduction of the effect of the urate-lowering allele of each pair. This effect was considerably stronger in women. For example, in the *SLC2A9 vs IRS1* analysis, serum urate increased by 1.6 μmol/L with the addition of 2-copies of the *IRS1* urate-decreasing allele within the GG (urate-lowering) genotype group at *SLC2A9* (Table 1).

**TABLE 1:** Association of the *SLC2A9* locus vs each of the *IRS1* / *IGF2* / *INSR* loci with serum urate levels stratified by sex and by genotype in the United Kingdom Biobank.

		*SLC2A9 (rs4447862)* C = urate-raising							
		Women (Δurate μmol/L., (SE))				Men (Δurate μmol/L, (SE))			
		CC	GC	GG		CC	GC	GG	
*IRS1 (rs61009233)* A = urate-raising	AA	Ref	−24.2 (0.4)	−63.2 (0.7)	**−*63.2***	Ref	−13.1 (0.5)	−36.6 (0.9)	**−*36.6***
	GA	−1.0 (0.4)	−24.6 (0.4)	−63.2 (0.8)	**−*62.2***	−0.3 (0.5)	−13.4 (0.5)	−38.3 (1.0)	**−*38.0***
	GG	−1.4 (0.6)	−25.7 (0.7)	−61.6 (1.5)	**−*60.2***	−0.1 (0.8)	−14.8 (0.9)	−36.7 (1.9)	**−*36.6***
		**−*1.4***	**−*1.5***	***+1.6***		**−*0.1***	**−*1.7***	**−*0.1***	
*IGF2 (rs3550608)* G = urate-raising	GG	Ref	−24.0 (0.3)	−62.7 (0.6)	**−*62.7***	Ref	−13.5 (0.4)	−37.5 (0.8)	**−*37.5***
	AG	−1.9 (0.4)	−25.4 (0.4)	−63.8 (0.9)	**−*61.9***	−1.7(0.5)	−14.5 (0.5)	−38.7 (1.1)	**−*37.0***
	AA	−5.0 (1.0)	−28.9 (1.2)	−64.2 (2.6)	**−*59.2***	−2.9 (1.3)	−15.1 (1.4)	−38.8 (3.2)	**−*35.9***
		−5.0	−4.9	−1.5		−2.9	−1.6	−1.3	
*INSR (rsl0405423)* A = urate-raising	AA	Ref	−24.1 (0.4)	−63.5 (0.8)	**−*63.5***	Ref	−12.6 (0.5)	−36.7 (1.0)	**−*36.7***
	CA	−1.9 (0.4)	−25.9 (0.4)	−63.9 (0.8)	**−*62.0***	−1.0 (0.5)	−14.8 (0.5)	−39.0 (0.9)	**−*38.0***
	CC	−4.3 (0.6)	−27.7 (0.7)	−65.1 (1.5)	**−*60.8***	−3.6 (0.7)	−17.3 (0.8)	−39.9 (1.8)	**−*36.3***
		**−*4.3***	**−*3.6***	**−*1.6***		**−*3.6***	**−*4.7***	**−*3.2***	

*The bolded italicized numbers at the end of each 3 genotype row and bottom of each 3 genotype column represent the change in urate according to *SLC2A9* genotype across rows and change in urate according to *IRS1 / IGF2 / INSR* genotype down columns.*

### Insulin Stimulates Urate Uptake Mediated by Endogenous Urate Transporters in Human Proximal Tubule Epithelial Cells (PTC-05) and HEK 293T Cells

**FIGURE 2:**
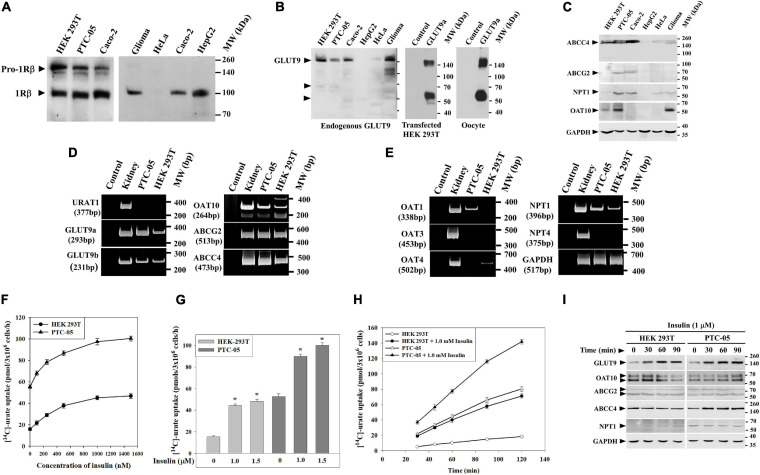
Insulin stimulates **[**^14^C**]**-urate uptake in human renal proximal tubule epithelial cells (PTC-05) and HEK 293T cells. **(A)** Western blot analyses show endogenous INSR protein (IRβ; ∼96 kDa) expression in various human cell lines. **(B)** Western blot analyses show endogenous GLUT9 protein expression in various human cell lines in transiently transfected (GLUT9a) HEK 293T cells and *Xenopus laevis* oocytes. **(C)** Western blot analyses show endogenous ABCC4, ABCG2, OAT10 and NPT1 protein expression in various human cell lines. **(D,E)** RT-PCR detection of the mRNA expression of URAT1, GLUT9 isoforms (GLUT9a and GLUT9b), OAT10, ABCG2, ABCC4, OAT1, OAT3, OAT4, NPT1, NPT4 and GAPDH in human kidney, PTC-05 and HEK 293T cells. **(F)** Dose-response plots showing the total [^14^C]-urate uptake activities of endogenous urate transporters, in equal number (3 × 10^6^) of HEK 293T or PTC-05 cells, in response to increasing concentration of insulin (0–1500 nM). **(G)** Bar diagram indicating the [^14^C]-urate uptake activities of endogenous urate transporters in HEK 293T and PTC-05 cells measured in the absence or presence of insulin (1.0 and 1.5 μM). Asterisk (“*”), *P* < 0.001 compared with urate uptake in the absence of insulin. **(H)** Time-course plot of insulin-stimulation of [^14^C]-urate uptake by endogenous urate transporters in HEK 293T or PTC-05 cells. Results are the average of three independent experiments ± s.e.m. **(I)** Western blot analyses show the effect of extracellular insulin (1.0 μM) on expression of endogenous urate transporter proteins in HEK 293T or PTC-05 cells for varying time intervals (0–90 min).

The net excretion of urate in the human urine reflects the balance between urate reabsorption and secretion, each process mediated by a separate set of urate transporters within the proximal tubule (Figures 1A,B). To explore the role of insulin-signaling in urate transport (Figure 1C), we initially utilized two human cell lines, human proximal tubule epithelial cells (PTC-05) and HEK 293T cells that express INSR (Figure 2A) and multiple urate transporters (GLUT9a, GLUT9b, OAT10, NPT1, ABCG2 and ABCC4) (Figures 2B–E). We detected additional expression of OAT1 only in PTC-05 cells. We pre-incubated an equal number (3 ×10^6^) of PTC-05 or HEK 293T cells with insulin (0.2 μM to 1.5 μM) in serum-free and potassium-free isotonic medium (Cicirelli et al., 1988, 1990), followed by measurement of [^14^C]-urate uptake. These transport experiments revealed a significant stimulation of [^14^C]-urate uptake by insulin (1.0 μM) in HEK 293T (∼2.5-fold increase) and PTC-05 (∼1.8-fold increase) cells (Figures 2F,G), transport activity that was almost linear with time for at least for 90 min (Figure 2H). We also examined the effect of insulin (1.0 μM) on expression levels of urate transporters. In HEK293T cells insulin increased the expression level of GLUT9 protein (∼3.75-fold increase after 90 min) but reduced the expression of OAT10 protein (∼64% decrease after 90 min) (Figure 2I). In PTC-05 cells however, we found significant elevation of GLUT9 (∼5.0-fold increase) and OAT10 (∼2.5-fold increase) protein levels after 90 min of exposure to insulin (Figure 2I).

### Insulin-Stimulation of Urate Uptake in Human PTC-05 and HEK 293T Cells Requires Activation of IRS-1, Akt, p44/42 MAPK (ERK1/2) and p38 MAPK

**FIGURE 3:**
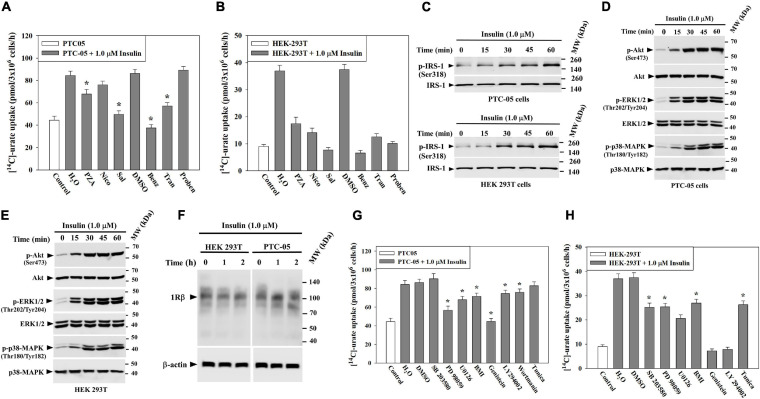
Insulin stimulates urate uptake through activation of PTK (protein tyrosine kinase) of INSR, IRS1, PI3K, Akt, p44/42 MAPK (ERK) and/or p38 MAPK in human kidney cell lines (HEK 293T and PTC-05). **(A,B)** The stimulatory effect of insulin (1.0 μM) on [^14^C]-urate uptake by endogenous urate transporters in equal number (3x10^6^) of PTC-05 **(A)** and HEK 293T **(B)** cells was measured in the absence and presence of 10 mM pyrazinoate (PZA), 10 mM nicotinate (Nico), 10 mM salicylate (Sal), 100 μM benzbromarone (Benz), 100 μM tranilast (Tran), or 1.0 mM probenecid (Proben) in the extracellular K^+^-free isotonic medium at ∼25°C. Uricosuric drugs were dissolved in dimethyl sulfoxide (DMSO). **(C–E)** Insulin activates IRS1, Akt, ERK and p38 MAPK in PTC-05 cells **(C,D)** and HEK 293T cells **(C,E)**. **(F)** Insulin has no significant effect on the expression of endogenous INSR protein (IRβ; **∼**96 kDa) expression. For Western blot analyses cells were treated with insulin (1.0 μM) in K^+^-free isotonic medium and lysates were analyzed using appropriate antibodies (see methods). **(G,H)** Signaling pathway-specific inhibitors differentially inhibit insulin-stimulation of [^14^C]-urate uptake in PTC-05 cells **(G)** and HEK 293T cells **(H)**. The effect of insulin (1.0 μM) on [^14^C]-urate uptake was measured in K^+^-free isotonic uptake medium in the absence and presence of the natural protein tyrosine kinase inhibitor (genistein; 75 μM), phosphatidylinositol 3-kinase (PI3K) inhibitor (LY 294002; 50 μM), wortmanin (4 μM), p38 MAPK inhibitor (10 μM SB 203580), MEK/ERK inhibitor (20 μM PD 98059 or U0126) or protein kinase C inhibitor (10 μM bisindolylmaleimide I (BMI). Results are the average of three independent experiments ± s.e.m. Asterisk (*), *P* < 0.001 compared with urate uptake in the absence of uricosuric drugs, anti-uricosuric agents or in presence of insulin and DMSO.

To verify whether insulin-stimulation of [^14^C]-urate uptake in PTC-05 and HEK 293T cells occurs via urate transporters, we examined [^14^C]-urate uptake in presence of uricosuric drugs (benzbromarone, probenecid and tranilast) or anti-uricosuric agents (pyrazinoate (PZA), nicotinate and salicylate). We found that insulin-stimulation of urate uptake in PTC-05 cells was very efficiently inhibited by benzbromarone (∼100% inhibition), tranilast (∼70% inhibition) or salicylate (∼87% inhibition), less efficiently inhibited by PZA (∼42% inhibition) or nicotinate (∼20% inhibition), and not inhibited at all by probenecid (Figure 3A). In HEK-293T cells, the insulin-stimulation of urate uptake was very efficiently inhibited by benzbromarone (100 μM; ∼100% inhibition), tranilast (100 μM; ∼90% inhibition), probenecid (1.0 mM; ∼90% inhibition), PZA (10 mM; ∼70% inhibition), nicotinate (10 mM; ∼84% inhibition) or salicylate (10 mM; ∼100% inhibition) (Figure 3B).

To characterize the signaling mechanisms whereby insulin stimulates urate uptake, we treated PTC-05 or HEK 293T cells with insulin (1.0 μM) for varying intervals of time (0 to 60 min) and analyzed the cell lysates for activation of IRS1, Akt, ERK1/2 or p38 MAPK (Figure 2C). The results show robust phosphorylation-dependent activation of IRS-1 (∼3.0/3.5-fold), Akt (∼6.2/4.2-fold), ERK1/2 (p44/42-MAPK) (∼10.6/8.7-fold) and p38 MAPK (∼8.2/5.5-fold) in PTC-05 (Figures 3C,D) or HEK 293T cells (Figures 3C,E) after exposure to insulin for 1h. Insulin treatment did not change INSRβ protein expression level in these cells (Figure 3F).

To verify whether insulin-stimulation of urate uptake in PTC-05/HEK 293T cells is dependent on the concurrent activation of IRS-1, Akt, ERK or p38 MAPK, we examined insulin stimulation of urate uptake in the presence of representative signaling pathway inhibitors (Supplementary Figure 2). The results show insulin-stimulation of urate uptake in PTC-05 cells was very efficiently inhibited (∼100% inhibition) in the presence of a protein tyrosine kinase (PTK) inhibitor (Genistein, 75 μM), but less efficiently inhibited in the presence a PI3K inhibitors (LY 294002, 50 μM, ∼28% inhibition; wortmannin, 4 μM, ∼27% inhibition) (Figure 3G), indicating that insulin-stimulation of urate transport requires activation of intrinsic protein tyrosine kinase (PTK) activity of the β-subunit of INSR, IRS-1 and PI3 kinase (PI3K) that leads to phosphorylation-dependent activation of Akt in PTC-05 cells (Figure 3D). In the presence of MEK/ERK inhibitor (PD 98059 or U0126, 20 μM) or protein kinase-C (PKC) inhibitor (10 μM bisindolylmaleimide 1), insulin-stimulation of urate transport was ∼70% or ∼35% inhibited, respectively (Figure 3G), however the p38 MAPK inhibitor (SB 203580, 10 μM) had almost no effect on insulin-stimulated urate transport. Therefore, in PTC-05 cells, the insulin-receptor seems to activate urate transport via the PI3K-PDK-1-Akt and Grb2-SOS-Ras-MAPK pathways.

In HEK 293T cells, the insulin-stimulation of urate uptake was almost completely inhibited in the presence of PTK or PI3K inhibitors (Figure 3H), and partially inhibited in the presence of MEK/ERK inhibitor (42–55% inhibition) (Figure 3H), p38 MAPK inhibitor (∼44% inhibition), or PKC inhibitor (∼35% inhibition) (Figure 3H). These results are consistent with a previous report in cultured cortical neurons (Duarte et al., 2005).

### Insulin Stimulates the Urate Transport Activity of GLUT9, OAT10, OAT3, OAT1 and NPT1

Since *Xenopus laevis* oocytes express a functional endogenous INSR (Stefanovic et al., 1986; Hainaut et al., 1991; Scavo et al., 1991) and have served as a model system for urate transport, we opted to exploit this system to identify the repertoire of human urate transporters that are activated by insulin signaling. Since the absence of extracellular K^+^ potentiates insulin-activated events in *Xenopus* oocytes (Cicirelli et al., 1988, 1990), we initially measured transport activity of each urate transporter in the absence or presence of extracellular Na^+^ or K^+^ ions. We expressed urate transporters in *Xenopus laevis* oocytes (Supplementary Figures 3A,B) by microinjecting equal picomoles of *in vitro* synthesized cRNA (see section “MATERIALS AND METHODS”).

Before examining the effect of insulin on urate transport activity of each urate transporter expressed individually in *Xenopus* oocytes, we assessed their urate transport activity in isotonic medium in the presence or absence of K^+^ or Na^+^ ions (Supplementary Figure 4A). In Na^+^-free medium (with 98 mM K^+^), the cell membrane of oocytes becomes depolarized; this depolarization stimulates urate uptake by GLUT9 isoforms (Supplementary Figure 4A). Notably, in K^+^-free medium the urate uptake activity of GLUT9b was 2.7-fold higher than that of GLUT9a (Supplementary Figure 4A) whereas in ND96 medium (2 mM K^+^, 96 mM Na^+^; pH 7.4), their urate transport activity remained almost equal (Supplementary Figure 4A), suggesting the N-terminal cytoplasmic domain of GLUT9a might sense extracellular K^+^ ion for its optimal urate transport activity.

**FIGURE 4:**
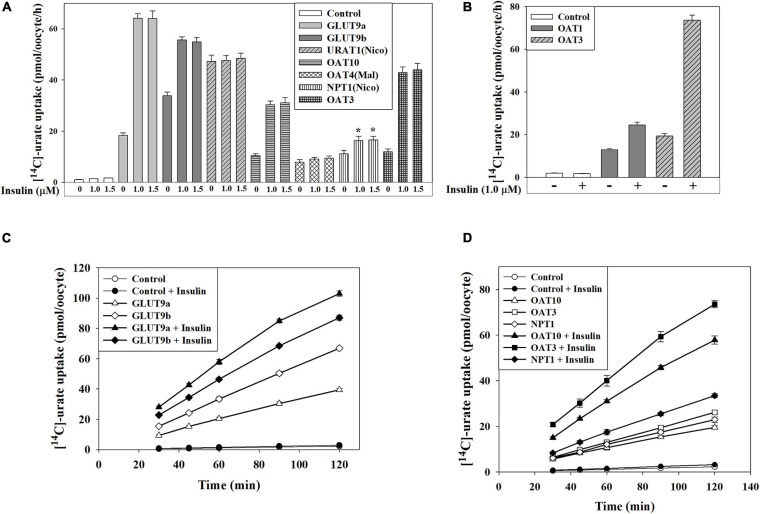
Insulin stimulates urate transport mediated by GLUT9, OAT10, OAT1, mOAT3 or NPT1 expressed in *Xenopus* oocytes. **(A,B)** The [^14^C]-urate uptake activity of human GLUT9 isoforms, URAT1, OAT10, OAT4, NPT1, OAT1, and mouse OAT3 was measured in absence and presence of insulin (1.0-1.5 μM) in K^+^-free isotonic medium. **(C,D)** The time course plot of [^14^C]-urate uptake mediated by human GLUT9a, GLUT9b, OAT10, NPT1 or mouse OAT3, expressed in oocytes, in the absence and presence of 1.0 μM insulin. Oocytes expressing URAT1, OAT10 or NPT1 were preloaded with 50 nl of 100 mM nicotinate (Nico) and oocytes expressing OAT4 were preloaded with maleate (Mal) with 50 nl of 100 mM maleate by microinjection 2 h before urate uptake. Asterisk (*), *P* < 0.001 compared with urate uptake/efflux in the absence of insulin.

We subsequently examined the effect of insulin on transport activity of each urate transporter. In ND96 medium (pH 7.4), insulin (1.0 μM) caused ∼25% increase of the activity of GLUT9b (Supplementary Figure 4B) without any significant effect on GLUT9a and URAT1 (Supplementary Figure 4B). In Na^+^-free isotonic medium (with 98 mM K^+^), the urate uptake activity of both GLUT9a and GLUT9b was about 15–16% increased by insulin, without any effect on URAT1 (Supplementary Figure 4C). However, in K^+^-free isotonic medium, wherein INSR signaling is maximized (Cicirelli et al., 1988; Cicirelli et al., 1990), insulin caused dramatic stimulations of the urate uptake activity of GLUT9a (∼3.5-fold increase), GLUT9b (∼1.7-fold increase), OAT10 (∼3-fold increase), OAT3 (∼4-fold increase), OAT1 (∼1.7-fold increase) and NPT1 (∼1.6-fold increase) (Figures 4A,B) without any effect on URAT1 and OAT4 (Figure 4A). Insulin even at very low concentration (50 nM) was found to significantly stimulate both GLUT9a and GLUT9b in K^+^-free medium, with a significant dose-dependency (Supplementary Figure 4D). The stimulation of [^14^C]-urate uptake by insulin was linear over time (Figures 4C,D) at least for 90 min. The urate efflux activity of GLUT9a/GLUT9b was not significantly affected by insulin (Supplementary Figures 5A,B), potentially due to simultaneous re-uptake of effluxed [^14^C]-urate by the insulin-activated, high-capacity transporters.

### Insulin Stimulates the Activity of Multiple Urate Transporters via Akt and ERK Pathways

**FIGURE 5:**
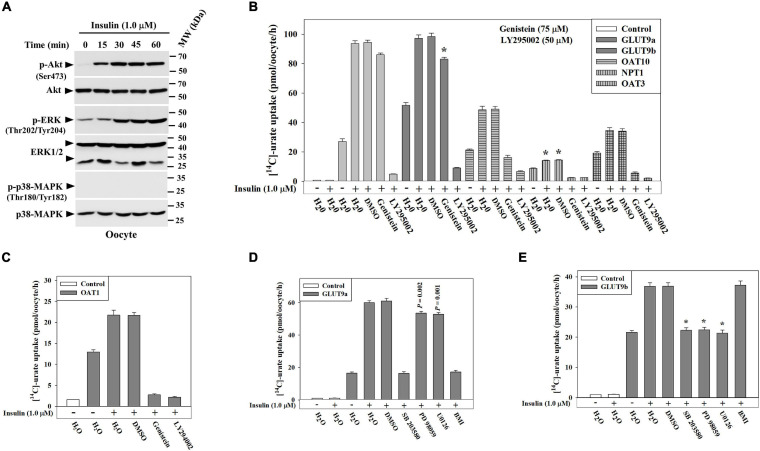
Insulin stimulates urate transport activity of GLUT9 isoforms, OAT10, NPT1, OAT3 or OAT1 in *Xenopus* oocytes via activation of protein tyrosine kinase (PTK) activity of INSR, phosphatidylinositol 3-kinase (PI3K), Akt, and/or p44/42 MAPK (ERK). **(A)** Western blot analyses of the lysates of *Xenopus* oocytes, treated with insulin (1.0 μM) at ∼25°C in K^+^-free isotonic medium using appropriate antibodies for indicated antigens. **(B,C)** The stimulatory effect of insulin (1.0 μM) on [^14^C]-urate uptake mediated by GLUT9 isoforms, OAT10, NPT1, mouse OAT3 and OAT1 in *Xenopus* oocytes was measured in the absence and presence of the natural PTK inhibitor (genistein; 75 μM) or PI3K inhibitor (LY 294002; 50 μM). Oocytes expressing OAT10 or NPT1 were preloaded with nicotinate (Nico) by microinjection of 50 nl of 100 mM nicotinate 2h before urate uptake. **(D,E)** Insulin-stimulation of urate uptake activities of human GLUT9a or GLUT9b was examined in oocytes in the absence and presence of p38 MAPK inhibitor (SB 203580; 10 μM), MEK/ERK inhibitor (PD 98059/U0126; 20 μM) or protein kinase C inhibitor (bisindolylmaleimide I hydrochloride, or BMI; 10 μM) in K^+^-free isotonic medium. Asterisk (*), *P* < 0.001 compared with urate uptake in the presence of insulin and DMSO.

To identify the intracellular signaling pathways that transmit signals from the INSR to urate transporters in *Xenopus* oocytes, we analyzed the lysates of oocytes treated with insulin (1.0 μM) in K^+^-free medium, looking for downstream phosphorylation events. Results of Western blotting shows about 15.5-fold activation of Akt (Ser-473), and 4.3-fold activation of p44/42 MAPK(Thr202/Tyr204) (ERK) in *Xenopus oocytes* in response insulin (Figure 5A) but failed to detect activation of p38 MAPK (Thr180/Tyr182) (Figure 5A) indicating the phospho-specific antibody might not recognize the phosphorylated *Xenopus* ortholog of p38 MAPK.

To verify whether the activation of Akt and/or ERK is required for functional activation of GLUT9 isoforms, OAT10, OAT3, OAT1 or NPT1 in oocytes, we measured insulin-stimulation of urate transport activity of each of these urate transporters in the absence and presence of a PTK-specific inhibitor (Genistein) or a PI3K-specific inhibitor (LY 294002). We found that the PI3K-specific inhibitor almost completely inhibited the insulin-stimulation of urate uptake mediated by GLUT9 isoforms, OAT10, OAT3, OAT1 and NPT1 (Figures 5B,C). PI3K inhibition also inhibited their basal urate transport activities (Figures 5B,C) suggesting the basal urate transport activities of these transporters require low level activation of PI3K. However, PTK inhibition with Genistein failed to significantly inhibit the insulin-stimulation of urate uptake by GLUT9 isoforms (Figure 5B), yet very efficiently inhibited (∼100% inhibition) the insulin-stimulation of urate uptake mediated by OAT10, OAT3, OAT1 and NPT1 (Figures 5B,C). Genistein also inhibited the basal urate transport activity of OAT3 (73% inhibition) (Figure 5B) and NPT1 (83% inhibition) (Figure 5B) while barely affecting the basal urate transport activity of OAT10 (Figure 5B).

We previously noticed differential stimulation of urate transport activity of GLUT9 isoforms by insulin (Figures 4A,C). To examine signaling pathway-specific differential regulation, we assayed the insulin-stimulation of urate transport activity of GLUT9 isoforms in the presence of downstream signaling pathway-specific inhibitors. Intriguingly, we found that in the presence of a MEK/ERK inhibitor, the insulin-stimulation of urate uptake by GLUT9a was about 12% inhibited whereas that by GLUT9b was almost completely inhibited. However, in the presence of a p38 MAPK-specific inhibitor, the insulin-stimulation of urate uptake by GLUT9a and GLUT9b was almost completely inhibited (Figures 5D,E). Again, in the presence of PKC-specific inhibitor, the insulin-stimulation of urate uptake by GLUT9a was almost completely inhibited (Figure 5D) whereas that mediated by GLUT9b was almost unaffected (Figure 5E).

The basal urate uptake activity of GLUT9 isoforms was also found to be differentially inhibited. In the presence of a MEK/ERK inhibitor, the basal urate uptake activity of GLUT9a was about 38% inhibited and that of GLUT9b was about 29% inhibited (Supplementary Figures 5C,D). In the presence of a p38 MAPK inhibitor, the basal urate uptake activity of GLUT9a was about 22% inhibited and that of GLUT9b was about 50% inhibited (Supplementary Figures 5C,D). In the presence of a PKC-specific inhibitor, the basal urate uptake activity of GLUT9a was about 10% inhibited and that of GLUT9b was almost uninhibited (Supplementary Figures 5C,D).

### N-terminal Cytoplasmic Domains of GLUT9 Isoforms are Important but Not Essential for Functional Activation by Insulin-Signaling

**FIGURE 6:**
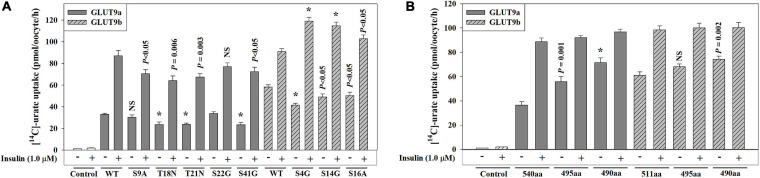
The N-terminal domain of human GLUT9 isoforms play a significant role in functional activation of by insulin. **(A,B)** The [^14^C]-urate uptake mediated by GLUT9a/GLUT9b, and its N-terminal point mutants **(A)** or its N-terminal deletion mutants **(B)** was examined in oocytes in the absence and presence of 1.0 μM insulin in K^+^-free isotonic medium (pH 7.4) at ∼25°C. Asterisk (*), *P* < 0.001 compared with the urate uptake by the normal urate transporter in the absence/presence of insulin.

The GLUT9 isoforms differ in their urate transport characteristics and their response to insulin-related signaling inhibitors (Supplementary Figures 4A,C, 5D,E Figure 4A). Since the human GLUT9 isoforms differ in their N-terminal cytoplasmic domains, with 50 unique residues for GLUT9a and 21 unique residues for GLUT9b, we set out to determine whether we could identify structural determinants for these physiological differences. We first examined the role of the unique N-terminal cytoplasmic domains by introducing single point mutations that convert the serine residues (S) into alanine (A) or glycine (G), and threonine (T) residues into asparagine (N), as S and T residues are the usual targets of phosphorylation by the kinases involved. The measurement of the urate transport activities of these mutants indicates that the [^14^C]-urate uptake activities of S9A, T18N, T21N, S22G and S41G mutants of GLUT9a were 90, 72, 72, 100, and 70%, respectively, of the basal urate transport activity of GLUT9a-WT and 81, 72, 77, 89, and 83%, respectively, of insulin-stimulated urate transport activity of GLUT9a-WT (Figure 6A). The S4G, S14G, and S16A mutants of GLUT9b exhibited 70, 83, and 87%, respectively, of the basal urate transport activity of GLUT9b-WT and 130, 127, and 113% of insulin-stimulated urate transport activity, respectively, of the GLUT9b-WT (Figure 6A). In summary, point mutations of possible phospho-acceptor sites within the N-terminal cytoplasmic domain of GLUT9a slightly reduced both its basal and insulin-stimulated activity;, mutations of sites within the N-terminal cytoplasmic domain of GLUT9b slightly attenuated its basal activity but significantly elevated its response to insulin.

Next, we examined whether their N-terminal cytoplasmic domains are at all required for their basal activity and for activation by insulin-signaling, by generating N-terminal deletion mutants. We thus generated GLUT9a mutants lacking 45 or 50 residues (GLUT9aΔ45; GLUT9aΔ50) and GLUT9b mutants lacking 16 or 21 residues (GLUT9bΔ16; GLUT9bΔ21) from their N-terminal ends. The measurement of the basal urate transport activities of GLUT9aΔ45 and GLUT9aΔ50 exhibited about 53% and 97% increase in K^+^-free medium, and about 13% and 22% decrease in ND96 medium (2 mM K^+^, 96 mM Na^+^) respectively, with respect to GLUT9a-WT (540 residues) (Figure 6B and Supplementary Figure 5E). The measurement of basal urate transport activities of GLUT9bΔ16 and GLUT9bΔ20 exhibited about 12% and 22% increase respectively in K^+^-free medium and about 28% increase and ∼19% decrease respectively in ND96 medium with respect to that of GLUT9b-WT (511 residues) (Figure 6B and Supplementary Figure 5E). The results demonstrate that the stretch of five amino acid residues (GRRRK in GLUT9a and AKKKL in GLUT9b) in the N-terminal cytoplasmic domains of GLUT9 isoforms determines their response to extracellular K^+^ ion. In the presence of insulin (1.0 μM), the [^14^C]-urate uptake activities of GLUT9a-WT, GLUT9aΔ45 and GLUT9aΔ50 were about 142, 66, and 35% increased, respectively (Figure 6B) and that of GLUT9b-WT, GLUT9bΔ16 and GLUT9bΔ20 were about 62, 46, and 34% increased, respectively (Figure 6B). Thus, the N-terminal cytoplasmic domains of the GLUT9 isoforms contribute partially to the response to insulin-signaling, without being completely essential.

### Signaling Pathways Required for the Functional Activation of OAT10, OAT3, OAT1, NPT1 and ABCG2 by Insulin

**FIGURE 7:**
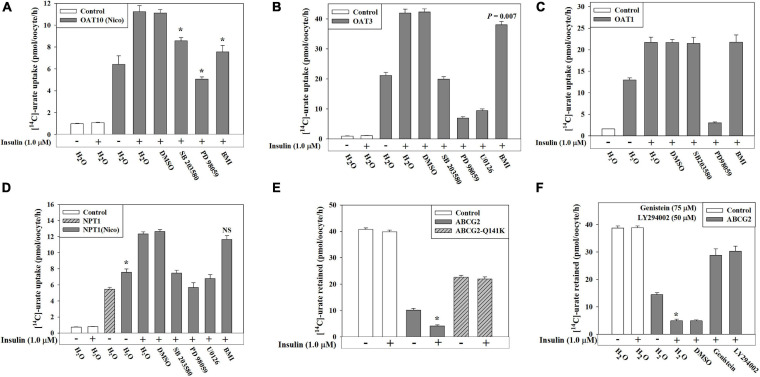
Insulin-stimulation of [^14^C]-urate uptake mediated by OAT10, OAT3, OAT1, NPT1 and ABCG2 in oocytes is inhibited by specific inhibitors of intracellular signaling pathways. **(A–D)** Insulin-stimulation of [^14^C]-urate uptake mediated by human OAT10 **(A)**, mouse OAT3 **(B)**, human OAT1 **(C)**, or human NPT1 **(D)**, expressed in oocytes, was measured in the absence or presence 1.0 μM insulin and p38 MAPK inhibitor (SB 203580; 10 μM), MEK/ERK inhibitor (PD 98059/U0126; 20 μM) or protein kinase C inhibitor (bisindolylmaleimide I hydrochloride, or BMI; 10 μM) in K^+^-free isotonic medium. Oocytes expressing NPT1 were preloaded with nicotinate (Nico) by microinjection of 50 nl of 100 mM nicotinate 2 h before urate uptake. **(E–F)** Insulin-stimulation of [^14^C]-urate efflux mediated by ABCG2 or ABCG2 mutant (Q141K) **(E)**, expressed in oocytes, was measured in the absence or presence 1.0 μM insulin and/or genistein (75 μM) or LY 294002 (50 μM) **(F)**, in K^+^-free isotonic medium (pH 7.4) at ∼25°C. Asterisk (*), *P* < 0.001 compared with the urate uptake by urate transporters in the absence/presence of insulin.

We demonstrated that PTK-specific inhibition and PI3K-specific inhibition almost completely inhibited insulin activation of the urate transport activity of human OAT10, human OAT1, murine OAT3 and human NPT1 expressed in oocytes (Figures 5B,C). To characterize the signaling pathway components further downstream of the INSR, we examined the insulin-stimulation of urate transport activity of these transporters in the presence of specific inhibitors of MEK/ERK (20 μM PD 98059), p38 MAPK (10 μM SB 203580), or PKC (10 μM bisindolylmaleimide 1 or BM1). We found that the insulin-stimulation of urate transport activities of OAT10, OAT1, mOAT3 and NPT1 were completely inhibited by the MEK/ERK inhibitor, about 55, 0, 100, and 100%, respectively, inhibited by the p38 MAPK inhibitor, and about 75, 0, 0, and 11%, respectively inhibited by the PKC inhibitor (Figures 7A–D). In addition, the basal urate transport activities of OAT10, mOAT3 and NPT1 was about 29%, 80%, and 44% inhibited by the MEK/ERK inhibitor, about 7, 0, and 25, respectively inhibited by the p38 MAPK inhibitor and about 7, 5, and 0%, respectively inhibited by the PKC inhibitor (Supplementary Figures 6A–C).

ABCG2 is an ATP-dependent export pump for urate (Woodward et al., 2009; Matsuo et al., 2011; Nakayama et al., 2011; Mandal et al., 2017). In this study, we found an approximately 18% increase in the urate efflux activity of ABCG2 in the presence of extracellular insulin (1.0 μM) in oocytes (Figure 7E and Supplementary Figures 6D,E). However, insulin did not have any significant effect on the ABCG2 hyperuricemia-associated mutant, Q141K (Woodward et al., 2009; Figure 7E), which exhibits only 50% of the urate transport activity of wild type ABCG2 (Figure 7E). The insulin-stimulation of ABCG2-mediated urate efflux was completely inhibited by PTK inhibitor or PI3K-specific inhibitor (Figure 7F) suggesting insulin-stimulation of the activity of ABCG2 works through activation of PTK activity of the INSR and through PI3K. In addition, the basal urate transport activity of ABCG2 was 40–45% affected in the presence of these inhibitors suggesting that the basal activity of ABCG2 requires low-level activation of both PTK and PI3K.

### Insulin Selectively Activates the Urate Transport Function of OAT10 Without Affecting Its Nicotinate Transport Function

We have previously shown about 3-fold increase in the urate uptake activity of the human OAT10, expressed in oocytes, in response to insulin (Figures 4A, 5B). Since OAT10 is also a high affinity nicotinate transporter (Bahn et al., 2008; Mandal et al., 2017), we examined the effect of insulin on its nicotinate transport activity. Surprisingly, we found almost no effect of insulin on [^14^C]-nicotinate uptake by OAT10 (Supplementary Figure 7) suggesting that the regulatory action of insulin is to selectively regulate the urate transport function of OAT10 without affecting its nicotinate transport function. We also examined the effect of insulin (1.0 μM) on nicotinate transport mediated by SMCT1, SMCT2 and URAT1 (Mandal et al., 2017), but did not find any noticeable effect of insulin on their nicotinate transport activities (Supplementary Figure 7).

## Discussion

The metabolic syndrome and type 2 diabetes are strongly associated with hyperuricemia and gout (Choi and Ford, 2007; Choi et al., 2007, 2008). It is well established that serum insulin levels correlate with SU (Quinones Galvan et al., 1995; Gill et al., 2013; MacFarlane et al., 2015; Wang et al., 2017) and insulin in euglycemic hyperinsulinemia, induced by euglycemic clamp protocols, reduces renal fractional excretion of urate (Quinones Galvan et al., 1995; Muscelli et al., 1996; Ter Maaten et al., 1997). Additionally, genetic variation in pathways of insulin signaling and energy metabolism is associated with variation in SU (Supplementary Figure 1; Köttgen et al., 2013; Phipps-Green et al., 2016; Tin et al., 2019). However, mechanistic information on the role of insulin in human urate homeostasis is sparse. To begin to clarify these associations we embarked on a genetic and physiological study of the role of insulin in urate transport regulation.

In this study we present evidence of genetic association of variants at the insulin, INSR and IRS-1 loci with SU using publicly-available data. By co-localizing the genetic signals with genetic control of gene expression, we and others (Tin et al., 2019) conclude that the genetic control is likely to be mediated by *IGF2* at the insulin locus, *INSR* and / or *ZNF358* at the *INSR* locus and *IRS1* at the *IRS1* locus (Supplementary Figure 1). The urate-raising allele at *INSR* correlates with increased INSR expression whereas the urate-raising locus at *IRS1* correlates with reduced IRS1 expression. In PTC-05 and HEK 293T cells, we found that insulin exposure activates IRS1 by phosphorylation without affecting its expression (Figure 3C). To the extent that secretory urate transporters are sensitive to genistein whereas GLUT9 is comparatively resistant (Figures 5B,C, 7F), we speculate that urate secretion is more sensitive to reduced IRS1 expression; that would then lead to a relative imbalance between urate flux across the proximal tubule with reduced IRS-1 availability, favoring reabsorption over secretion (Figure 1).

Collectively these genetic data are consistent with a causal role for insulin in control of serum urate levels. Further supporting evidence for this could be provided by formal Mendelian randomization studies using a systematically identified suite of genetic variants associated with insulin resistance (and signaling phenotypes) testing for a causal role in serum urate levels. Epistatic interaction of *SLC2A9* with genetic variation at the *IRS1, IGF2* and *ZNF358* loci further supports a causal role of insulin signaling in the control of serum urate levels in humans. Epistatic interactions were stronger in women, consistent with the greater genetic effect of *SLC2A9* on serum urate levels in women (Köttgen et al., 2013). Overall, this genetic interaction between *SLC2A9* and insulin-related genes supports the primacy of insulin-activation of GLUT9 in the anti-uricosuric effect of insulin (see below). However, given the multiple genetic influences on systemic urate homeostasis, involving multiple pathways of metabolism and physiology (Tin et al., 2019), we expect that insulin influence on urate homeostasis is not limited to effects on urate transporters.

To establish a functional link between insulin and urate transport (Figure 1C), we first examined the effects of insulin on expression and activity of urate transporters in human proximal tubule epithelial cells (PTC-05, representative of proximal tubule physiology) (Orosz et al., 2004) and HEK 293T cells (a widely utilized, easily transfectable human cell line) (Figures 2F-I). These cells have distinct repertoires of endogenous urate transporters and provided a duplicate comparator for the transport and signaling effects that we detected in *Xenopus* oocytes. We found that insulin markedly stimulated urate uptake mediated by endogenous urate transporters in PTC-05 and HEK 293T cells (Figures 2F–H) and also increased the expression levels of GLUT9 and OAT10 in PTC-05 cells; only GLUT9 protein expression was induced by insulin in HEK 293T cells (Figure 2I). Moreover, we found that the insulin-stimulation of urate uptake, associated with the concurrent activation of IRS1, Akt, MEK/ERK and/or p38 MAPK signaling pathways (Figure 3C–E), was effectively inhibited by the specific inhibitors of these signaling pathways (Figure 3G,H). Therefore, these results suggest a regulatory role of insulin signaling on the expression and/or activities of human urate transporters, particularly GLUT9 and OAT10. The limitations of studying net uptake in human cell lines under non-polarized conditions are acknowledged. For individual urate transporters *in vivo* the magnitude and direction of transport is dictated by activity of the transporter, the gradient of urate, membrane and transcellular potentials, and for some of the transporters the intra- and extracellular concentration of exchanging substrates (“cis-inhibition” and “trans-activation”) (Mandal and Mount, 2015).

The complete inhibition of insulin-stimulation of urate uptake by PTK inhibitor or PI3K inhibitor in HEK 293T cells (Figure 3H) and the almost complete inhibition by PTK inhibitor and partial inhibition by PI3K inhibitor (∼27.4%) in PTC-05 cells (Figure 3G) suggests that insulin-stimulation of urate uptake is regulated by insulin-INSR signaling through PI3K and Akt activation. In PTC-05 and HEK 293T cells the varying degrees of inhibition of urate transport by specific inhibitors of MEK/ERK, p38MAPK or PKC (Figures 3G,H) suggest involvement of multiple signaling components branching out from the INSR, likely affecting multiple urate transporters. The potential for off-target effects of the pharmacological inhibitors utilized is acknowledged; however, these inhibitors did predictably block insulin-activated phosphorylation of IRS-1, Akt, and/or Erk, providing internal confirmation of their primary reported effects (Supplementary Figure 2).

By exploiting the functional insulin receptor of *Xenopus laevis* oocytes (Stefanovic et al., 1986; Hainaut et al., 1991; Scavo et al., 1991), a key heterologous expression system for urate transporters, we identified six urate transporters (GLUT9, OAT10, OAT3, OAT1, NPT1 and ABCG2) that are regulated by insulin signaling (Figures 4A–D, 5B,C). Again, with the exception of ABCG2, our oocyte experiments were primarily limited to uptake assays as an indicator of transporter activity; the direction of transport *in vivo* depends on multiple physiological influences that are unique to each cell type and transporter (Mandal and Mount, 2015). We found that insulin at very low concentration (50 nM) significantly stimulated the activity of both GLUT9a and GLUT9b in K^+^-free medium (Supplementary Figure 4D). We also found that insulin triggers the activation of Akt (Ser473) and ERK (Thr202/Tyr204) in oocytes, analogous to the effect of insulin in human cell lines (Figure 5A). Importantly, in oocytes the insulin-stimulation of urate uptake activities of GLUT9 isoforms, OAT10, OAT3, OAT1, NPT1 and ABCG2 was almost completely abolished in the presence of a PI3K inhibitor (Figures 5B,C, 7F), in addition to significant inhibition of their basal urate transport activities; this suggests a key role for PI3K and downstream kinases, including Akt, in the regulation of urate transport. Therefore, targeting this signaling pathway may prove effective for the treatment of hyperuricemia associated with gout and metabolic syndrome.

Intriguingly, we found considerable heterogeneity in the response of insulin-sensitive urate transporters to other signaling pathway-specific inhibitors. Genistein, a potent PTK inhibitor, failed to efficiently inhibit the insulin-stimulation of urate transport mediated by GLUT9 isoforms (∼12% inhibition for GLUT9a and ∼33% inhibition for GLUT9b), yet very effectively inhibited the insulin-stimulated urate transport activities of OAT10, OAT3, OAT1, NPT1 and ABCG2 (Figures 5B,C, 7F), along with a significant effect on their basal urate transport activities. MEK/ERK inhibition almost completely inhibited the insulin-stimulation of urate uptake mediated by GLUT9b, OAT10, OAT3, OAT1 and NPT1 (Figures 5E, 7A–D). Inhibition of p38 MAPK almost completely inhibited the insulin-stimulation of urate uptake mediated by GLUT9a, GLUT9b, OAT3 and NPT1 (Figures 5D,E, 7B,D). PKC inhibition also almost completely inhibited the insulin-stimulation of urate uptake mediated by GLUT9a (but not GLUT9b) (Figures 5D,E) and ∼77% of the activity of OAT10 (Figure 7A). The collective results in the PTC-05 and HEK 293T cells and oocytes suggest involvement of multiple intracellular signaling components in transmitting signals from the insulin-INSR complex to urate transporters. Again, the potential for off-target inhibition of these various pharmacological agents is acknowledged; validation of direct effects on these multiple transporters will have to await characterization of the phosphorylation sites for each transporter and/or associated regulatory proteins.

This study also revealed four significant differences between the two major isoforms of GLUT9; the isoforms differ in their basal urate transport activity in K^+^ ion-free isotonic medium (Figure 4A and Supplementary Figure 4A), the extent of membrane potential-driven elevation of activity in Na^+^-free isotonic medium (Supplementary Figure 4A), the extent of activation by insulin (Figures 4A,C) and the inhibition characteristics of insulin-stimulated activity in the presence of inhibitors of PTK, MEK/ERK or PKC (Figures 5D,E). Mutation of the serine and threonine residue at phospho-acceptor sites unique to GLUT9a and GLUT9b partially affected functional activation by insulin-INSR signaling (Figure 6A). Deletion mutants of the N-terminal cytoplasmic domains of the GLUT9 isoforms showed enhanced basal activity with residual activation by insulin for both isoforms (Figure 6B), suggesting roles for the N-terminal cytoplasmic domain and other domains of GLUT9 in activation by insulin. We expect the differential regulation of the activity of GLUT9a and GLUT9b by insulin to be highly complex, analogizing to the molecular mechanisms involved in insulin regulation of the GLUT4 glucose transporter (Klip et al., 2019).

We did not find any effect of insulin on urate transport activities of URAT1 and OAT4 (Figure 4A). Additionally, we found that insulin selectively activates the urate transport activity of OAT10 (Figures 4A,D, 5B, 7A), without effect on its nicotinate transport activity (Supplementary Figure 7), indicating highly specific regulation of OAT10-mediated urate transport by insulin. Insulin also had no effect on nicotinate transport activities of other nicotinate transporters (SMCT1, SMCT2 and URAT1) (Supplementary Figure 7). This suggests a high degree of specificity for insulin-regulation of urate transport in preference to nicotinate transport.

We also found that insulin-activation of Akt and ERK was not significantly inhibited in the presence of 600 μM urate in PTC-05, HEK 293T cells or in oocytes (Supplementary Figure 8), indicating that urate does not inhibit insulin signaling in these cells. The limitations of these cellular systems for the study of systemic insulin resistance are acknowledged; however, these data fit with the results of Mendelian randomization studies indicating that urate homeostasis does not affect the development of type 2 diabetes (Johnson et al., 2018) or hyperinsulinemia (McCormick et al., 2021) in human subjects.

In summary, we have determined that insulin activates both “re-absorptive” (GLUT9 and OAT10) and “secretory” (OAT1, OAT3, NPT1 and ABCG2) urate transporters (Figures 4A,B, 7E,F). We have also determined that the basal transport activity of GLUT9 vastly exceeds that of other urate transporters (Supplementary Figure 4A). Additionally, the relative activation of urate transport activity of GLUT9a by insulin is much higher than other urate transporters (Figures 4A, 5B). To the extent that insulin reduces renal fractional excretion of urate (Quinones Galvan et al., 1995; Muscelli et al., 1996; Ter Maaten et al., 1997), it must activate renal re-absorptive urate transport to a greater extent than secretory transport. Given that GLUT9a is the sole transporter for basolateral exit of reabsorbed urate into blood (Kimura et al., 2014; Mandal and Mount, 2015) and renal insulin sensitivity remains preserved in hyperinsulinemia (Nizar et al., 2018), we propose that activation of GLUT9a by insulin is a key driver of the renal urate retention in metabolic syndrome associated with hyperuricemia and gout. This proposal is supported by our finding of epistatic interaction between *SLC2A9* and serum urate-associated genetic variants at each of the *IRS1, IGF2* and *INSR* loci, where the combination of urate-lowering alleles in each pair-wise comparison blunted their urate-lowering effect. Notably, however, much remains to be clarified regarding the molecular mechanisms involved in activation of GLUT9 and other urate transporters by insulin. Additionally, we expect that the impact of insulin resistance on these mechanisms will be complex, involving multiple other mediators (Horita et al., 2011).

Formal characterization of the *in vivo* role of GLUT9 in insulin-activated urate retention will require a study of the effect of insulin on human subjects with genetic variation in *SLC2A9*, given that GLUT9 is not expressed in the proximal tubule of mice (Preitner et al., 2009); single cell RNA-sequencing of mouse kidney indicates exclusive expression within distal connecting tubules (Wu et al., 2019). Hoque et al. recently reported the use of inosine loading to characterize the role of GLUT9 in human urate physiology, demonstrating a higher fractional excretion of urate (FeU) in the ∼30% of participants with the protective (urate-lowering) C allele of *SLC2A9* rs11942223 (Hoque et al., 2020). This indicates that the urate-lowering allele in *SLC2A9* reduces GLUT9 function in urate reabsorption within the kidney, providing an opportunity to examine the role of GLUT9 in the anti-uricosuric effects of insulin using a euglycemic clamp protocol (Quinones Galvan et al., 1995; Muscelli et al., 1996; Ter Maaten et al., 1997). Specifically, given the clear separation in FeU between *SLC2A9* genotypes, with a significant increase in subjects with the “urate-lowering” allele, *SLC2A9* genotype would be expected to affect the expected drop in FeU after insulin.

We postulate that GLUT9a is the major isoform involved in the anti-uricosuric effect of insulin, given that it serves as the sole basolateral exit pathway for urate for re-absorption across proximal tubule epithelia (Kimura et al., 2014). However, the GLUT9b isoform is also expressed in human kidney, at the apical membrane of cells in the collecting duct (Kimura et al., 2014). Of note, single cell RNA sequencing databases for human kidney indicate mRNA expression of URAT1, OAT10, GLUT9, OAT3, NPT4, ABCG2, and ABCC4 in intercalated cells of the distal nephron. Major unresolved issues include the role of these distal tubular urate transporters in urate homeostasis and the specific direction of their physiological transport *in vivo*. In particular, apical GLUT9b (Augustin et al., 2004; Kimura et al., 2014) in the distal nephron conceivably mediates apical, reabsorptive urate uptake in these cells; like GLUT9a, GLUT9b is activated significantly by insulin. Expression levels of GLUT9b in white blood cells also appear to demonstrate a greater association than GLUT9a with SU, suggesting unexplored roles for this isoform in apical urate transport by the human distal nephron and/or other aspects of urate homeostasis (Doring et al., 2008). Therefore, translational studies of the role of GLUT9b in the anti-uricosuric effect of insulin are also indicated, determining for example if the appearance of urinary GLUT9b exosomes, as an indirect index of apical trafficking (Kanno et al., 1995; Elliot et al., 1996), are affected by insulin in euglycemic clamp studies.

## Conclusion

Genetic variation in human insulin signaling impacts serum urate levels, with significant epistatic interaction in urate effects between insulin-related genes and the *SLC2A9* gene encoding GLUT9. Insulin activates several urate transporters, with more pronounced effects on GLUT9a, the exclusive basolateral exit pathway for urate reabsorption of urate in the proximal tubule; we postulate that the anti-uricosuric effect of insulin is primarily due to the enhanced expression and activation of GLUT9a.

## Data Availability Statement

The original contributions presented in the study are included in the article/Supplementary Material, further inquiries can be directed to the corresponding author/s.

## Ethics Statement

This research was conducted by using the UK Biobank resource (approval No. 12611). Ethical approval for the UK Biobank participants was obtained from the North West Multi-Centre Research Ethics Committee (11/NW/0382). All participants gave written informed consent. The animal study was reviewed and approved by the Brigham and Women’s Hospital.

## Author Contributions

DM, AM, HC, and TM conceived the study. AM performed and CE assisted in the transport and biochemical experiments. ML and TM analyzed genetic data. AM and DM wrote the manuscript draft. All authors contributed to completion and editing of the manuscript.

## Conflict of Interest

The authors declare that the research was conducted in the absence of any commercial or financial relationships that could be construed as a potential conflict of interest.

## Publisher’s Note

All claims expressed in this article are solely those of the authors and do not necessarily represent those of their affiliated organizations, or those of the publisher, the editors and the reviewers. Any product that may be evaluated in this article, or claim that may be made by its manufacturer, is not guaranteed or endorsed by the publisher.
