# Supplementary material for: Genetic and Physiological Effects of Insulin on Human Urate Homeostasis
Source: Front Physiol. 2021 Aug 2;12:713710. doi: 10.3389/fphys.2021.713710 (PMC8366499; doi:10.3389/fphys.2021.713710)
Supplement: Supplementary file 1 [file Data_Sheet_1.docx]

Supplementary Material

Genetic and Physiological Effects of Insulin on Human Urate Homeostasis

**Authors:** Asim K. Mandal^1^, Megan P. Leask^3,4^, Christopher Estiverne^1^, Hyon K. Choi^5^, Tony R. Merriman^3,4^, David B. Mount^1,2*^

**Affiliations:**

Renal Divisions,^1^Brigham and Women’s Hospital and ^2^VA Boston Healthcare System, Harvard Medical School,

Boston, MA

^3^Biochemistry Department, University of Otago, Dunedin, New Zealand

^4^Division of Rheumatology and Clinical Immunology, University of Alabama, Birmingham AL

^5^Division of Rheumatology, Massachusetts General Hospital, Harvard Medical School, Boston MA

***Correspondence:**Dr. David B. Mount, Associate Chief, Renal Division, Brigham and Women’s Hospital, Harvard Medical School, Room 540, 4 Blackfan Circle, Boston, MA 02115. Telephone: 617**-**525**-**5876 Fax: 617 732 6092 Email: dmount@bwh.harvard.edu

# Supplementary Data

**GLUT9 mutagenesis primers:**

Mutations in GLUT9 were introduced by site-directed mutagenesis PCR reaction using the QuickChange II site-directed mutagenesis kit (Agilent Technologies, Santa Clara, CA) following the manufacturer’s instructions. Primer sequences for introducing mutations into the N-terminal domains or designing constructs with deletion of N-terminal domains of GLUT9 isoforms are listed in Supplementary materials. Nucleotides mutated for experiments are shown bold and underlined. (S = sense; A = antisense)

**Primers for introducing point mutations into the N-terminal domains of GLUT9 isoforms:**

GLUT9a-S9A-1S **→** GGAAACAAAATAGGAAT**G**C**A**AAGGAACTGGGCCTAG

GLUT9a-S9A-1A **→** CTAGGCCCAGTTCCTT**T**G**C**ATTCCTATTTTGTTTCC

GLUT9a-T18N-1S **→** GCCTAGTTCCCCTCA**AC**GATGACACCAGCCAC

GLUT9a-T18N-1A **→** GTGGCTGGTGTCATC**GT**TGAGGGGAACTAGGC

GLUT9a-T21N-1S **→** CTCACAGATGACA**AT**AGCCACGCCGGGCCTC

GLUT9a-T21N-1A **→** GAGGCCCGGCGTGGCT**AT**TGTCATCTGTGAG

GLUT9a-S22G-1S **→** CACAGATGACACC**G**G**C**CACGCCGGGCCTC

GLUT9a-S22G-1A **→** GAGGCCCGGCGTG**G**C**C**GGTGTCATCTGTG

GLUT9a-S41G -1S **→** GACCACCTGAGG**G**G**C**GGGGTGCCAGGTGGAA

GLUT9a-S41G -1A **→** TTCCACCTGGCACCCC**G**C**C**CCTCAGGTGGTC

GLUT9b-S4G-1S **→** GAAAAGTGAACCATGAAGCTC**G**G**C**AAAAAGGACCGAGG

GLUT9b-S4G-1A**→** CCTCGGTCCTTTTT**G**C**C**GAGCTTCATGGTTCACTTTTC

GLUT9b-S14G-1S **→** ACCGAGGAGAAGATGAAGAA**G**G**C**GATTCAGCGAAAAAG

GLUT9b-S14G-1A **→** CTTTTTCGCTGAATC**G**C**C**TTCTTCATCTTCTCCTCGGT

GLUT9b-S16A-1S **→** GGAGAAGATGAAGAAAGTGAT**G**C**G**GCGAAAAAGAAATTGG

GLUT9b-S16A-1A **→** CCAATTTCTTTTTCGC**C**G**C**ATCACTTTCTTCATCTTCTCC

GLUT9b-S24A-1S **→** AGAAATTGGACTGG**G**C**G**TGCTCGCTCCTCGTG

GLUT9b-S24A-1A **→** CACGAGGAGCGAGCA**C**G**C**CCAGTCCAATTTCT

GLUT9b-S26A-1S **→** GGACTGGTCCTGC**G**CGCTCCTCGTGGCCTC

GLUT9b-S26A-1A **→** GAGGCCACGAGGAGCG**C**GCAGGACCAGTCC

Primers for generation of N-terminal deletion constructs of GLUT9 isoforms: (restriction sites are underlined and termination codon bold; S = sense; A = antisense)

GLUT9-24S **→** ACGCCCGGGATGGACTGGTCCTGCTCGCTCCTCGTGGCCTC

GLUT9a-25S **→** ACG CCCGGGATGGGAAGGAGAAGAAAGGACTGGTCCTGCTCGCTC

GLUT9b-25S **→** ACG CCCGGGATGGCGAAAAAGAAATTGGACTGGTCCTGCTCGCTC

GLUT9a-26S **→** ACGCCCGGGATGGCAAGGAAACAAAATAGGAATTCCAAGGAACTGG

GLUT9b-26S**→** ACGCCCGGGATGAAGCTCAGTAAAAAGGACCGAGGAGAAGATGAAGAAAGTG

GLUT9-27A --> GGAAGCTTG**TTA**AGGCGTTCCATTTATCTTACCATCAGTG

All introduced mutations were confirmed by sequencing.

**RT-PCR primers:**

Total RNA from PTC-05 and other cells as indicated was extracted using spin columns with the RNeasy Mini Kit (QIAGEN, GmbH, Germany) following the manufacturer’s instructions. About 2 μg of total RNA, isolated from cells, were primed with poly-dT and random hexamers and then reverse-transcribed using AMV reverse transcriptase (New England Biolabs, Ipswich, MA). Equal amount of cDNA was used for PCR amplification keeping a negative control lacking template cDNA. Primers utilized for RT-PCR are listed below. All PCR products were confirmed by cloning and sequencing.

The following intron-spanning primers were used for PCR amplification: (S = sense; A = antisense)

URAT1-4S → 5’-GGTTCCAGGTTCTCCAGACG-3’

URAT1-4A → 5’-GTCACACACGAGGTTCCACT-3’

GLUT9a-10S → 5'-AATGCCTTGGCAGAGTCTGG -3'

GLUT9a-1A **→** 5'-CACCACCGACAGGTTGTAGCCGTAG-3'

GLUT9b-S1 → 5’-CCATGAAGCTCAGTAAAAAGGAC-3’

GLUT9b-A1 → 5’-GAGTGTCTGGGTCTATTGGACG-3’

OAT10-S5 → 5’-AATGAGACGCAGCCTTGTGA-3’

OAT10-A5 → 5’-GTAGAGCTCAAAGCTGGGCA-3’

ABCG2-S1 → 5’-AGCTGCAAGGAAAGATCCA-3’

ABCG2-A1 → 5’-AGTCTTCCTGAGGCCAATAAG-3’

ABCC4-S2 → 5’-AGTTGGTTTTCAGGCCTATAAG-3’

ABCC4-A2→ 5’-CACCAACCACTTGTAGCAATG-3’

OAT1-S1 → 5’-CGCCGGAAGGTACTCATCTTG-3’

OAT1-A1 → 5’-CGGGCCGACTCAATGAAGAAC-3’

OAT3-S2 → 5’-GAGAAAGGCTCAGCTTGGAGGAGCTCAAACTCAA-3’

OAT3-A2 → 5’-TGACTGTGGGGTATAATTCACTTGTGTAGAGGAAGAGGC-3’

OAT4-1S → 5’-GCAAACCAGACCAAGCACTTCAGGAG-3’

OAT4-1A → 5’-TCAGCCTTGTAGATGGTGAGGCAGGT-3’

NPT1-S2 → 5’-CCAGCAGGTCAGTTCAAGTAG-3’

NPT1-A2 → 5’-GTTGCACCAGCAAGTATTAGG-3’

NPT4-S2 → 5’- GGTCTTCTAAGCAGCCTCTTC-3’

NPT4-A2 → 5’-AAGAGAGCGTCAGCAAGGCA-3’

GAPDH-1S → 5'-CGGAGTCAACGGATTTGGTCGTATTG-3'

GAPDH-1A → 5'-GACTGTGGTCATGAGTCCTTCCACGA-3'

All PCR products were confirmed by cloning and sequencing.

# Supplementary Figures


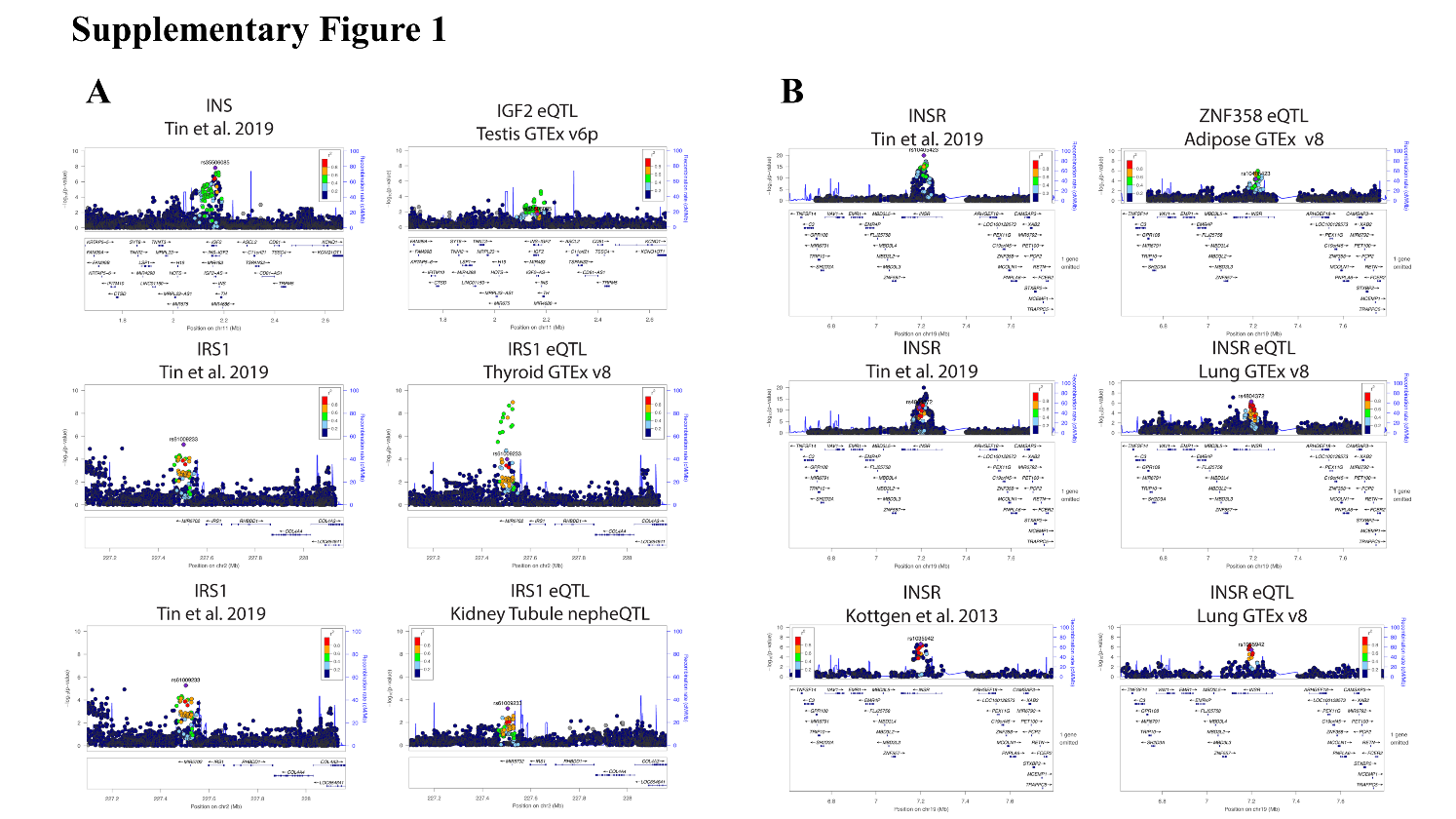


**Supplementary Figure 1**

**Locus Zoom plots of association of genetic variants with serum urate levels (left) and with gene expression (right).** Association data with serum urate levels was taken from Tin *et al* and used to generate the plots on the left. Genomic position is on the x-axis, -log_10_ *P* for association with urate is on each genetic variant is a single dot and linkage disequilibrium (LD) relationship with the lead (most associated) variant is indicated by the color of the dot (from strong LD (red) to weak / no LD (dark blue)). Association data with gene expression was taken from the Genotype-Tissue Expression database ([www.gtex.org](http://www.gtex.org)). Representative plots are shown. Note that the urate-associated signal at the *INSR* locus co-localized with expression of each of *INSR* and *ZNF358*.


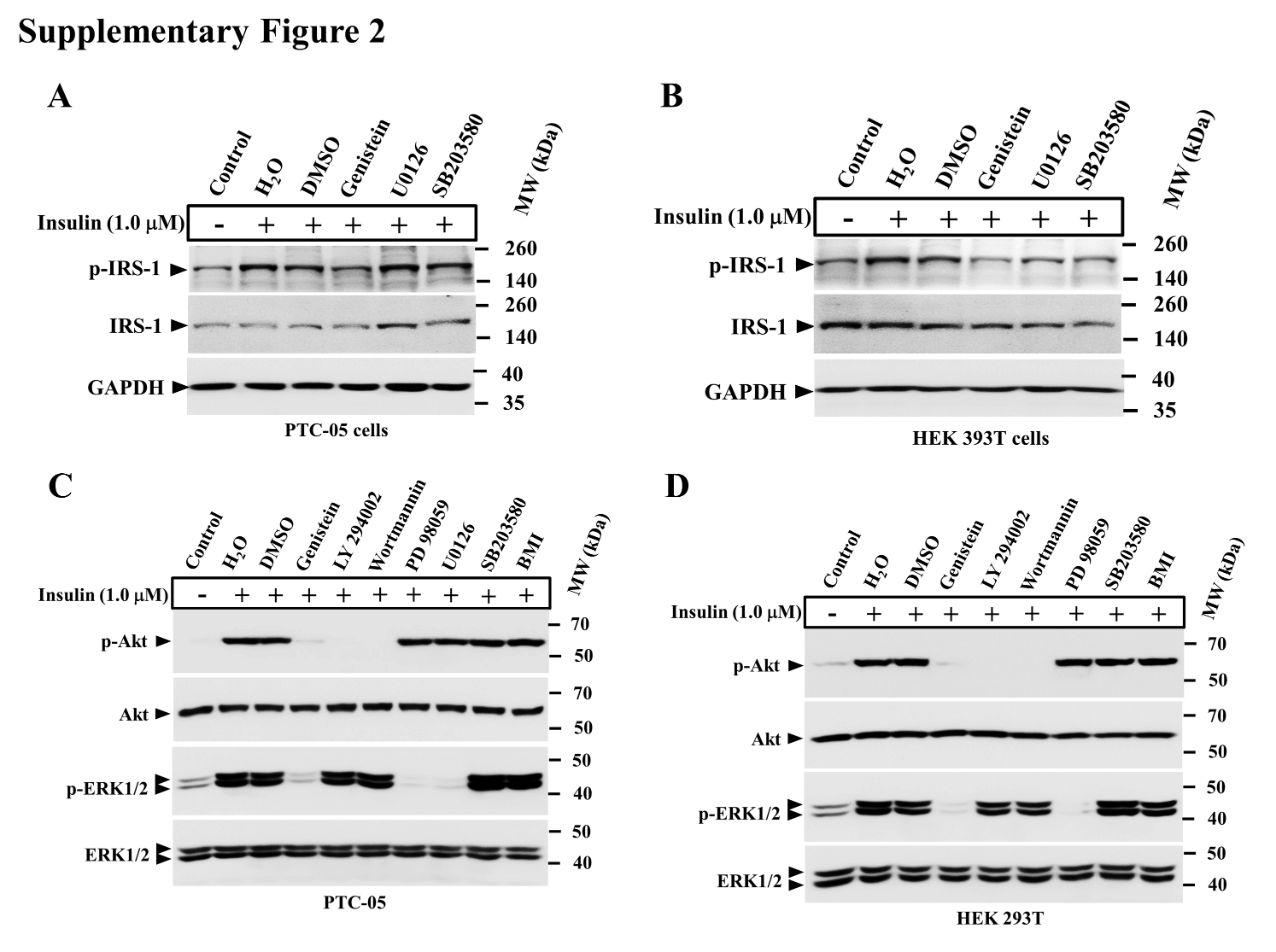


**Supplementary Figure 2**

Specificity of intracellular signaling pathway inhibitors: (**A** and **B**) Insulin receptor substrate 1 (IRS1) activation by insulin-signaling is inhibited in presence of protein tyrosine kinase (PTK) inhibitor, genistein (75 µM) in human renal proximal tubule epithelial cells (PTC-05) (**A**) and HEK 293T cells (**B**). (**C** and **D**) Activation of Akt by insulin signaling is inhibited in presence of PTK inhibitor, genistein, PI3K inhibitor, LY294002 (50 µM) or wortmannin (2 µM) in PTC-05 cells (**C**) and HEK 293T cells (**D**). Activation of ERK by insulin signaling is inhibited in presence of PTK inhibitor, genistein, MEK/ERK inhibitor PD98059 (20 µM) or U0126 (10 µM) in K^+^-free and serum-free isotonic medium in PTC-05 cells (**C**) and HEK 293T cells (**D**). Western blot analyses of cell lysates (30 µg/lane) of PTC-05 or HEK 293T cells treated without or with insulin (1.0 µM) in K^+^-free and serum-free isotonic medium (see methods) for 1h at room temperature (~25^0^C) in the absence or presence of DMSO (2.5 µl/ml), or indicated inhibitor using rabbit anti-phospho IRS1 (Ser318) antibody, anti-phospho-Akt (Ser473) antibody, anti-phospho-ERK1/2 (Thr202/Tyr204) antibody, anti-phospho p38 MAPK (Thr180/Tyr182) antibody, anti-IRS1 antibody, anti-Akt antibody, anti-ERK1/2 antibody or anti-p38 MAPK antibody. Proteins were fractionated using 8.0% SDS/PAGE. Each experiment shown here was performed at least three times for confirmation; data for each figure are from a single representative experiment.


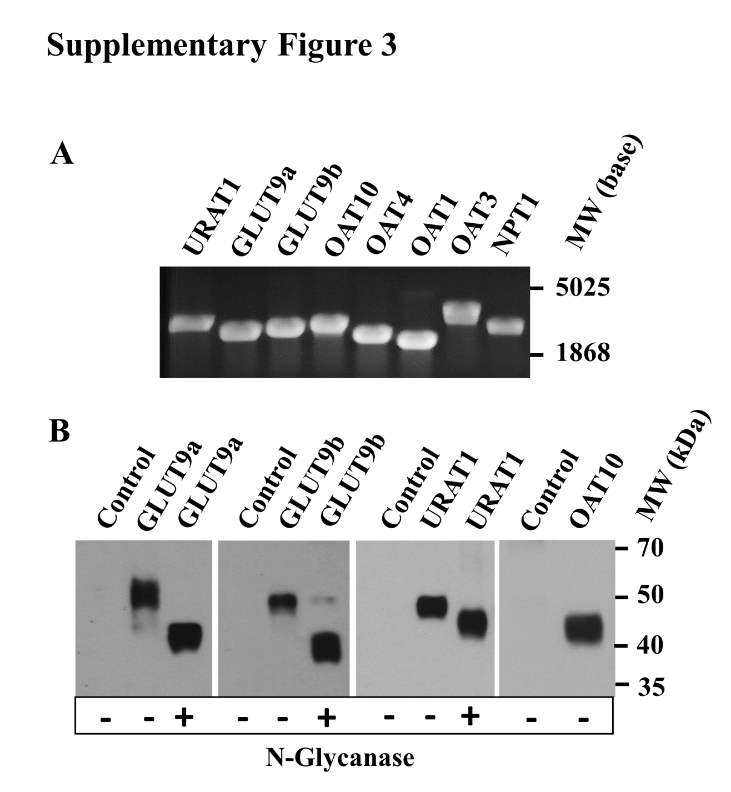


**Supplementary Figure 3**

Expression of human and mouse urate transporters in oocytes. (**3A)** Upper panel: *In vitro* synthesized cRNAs of urate transporters (URAT1, GLUT9a, GLUT9b, OAT10, OAT4, OAT1, mOAT3, and NPT1) were analyzed by 1.0% agarose-formaldehyde gel electrophoresis followed by ethidium bromide staining for checking their intactness before microinjection into each oocyte. (**3B)** Lower panel: Western blot analyses of the expression of urate transporter proteins (GLUT9a, GLUT9b, URAT1 and OAT10), in *Xenopus* oocytes after microinjection of their respective cRNA and incubation for 48 hours in ND96 medium supplemented with pyruvate (see methods), using rabbit anti-GLUT9, anti-URAT1 or anti-OAT10 antibody. Before Western blot analyses, oocyte extracts (expressing urate transporter protein) were digested with N-glycanase at 37^o^C for 4 hours as indicated to check their N-glycosylation status in oocytes. About 20 µg of the total proteins from oocyte lysates undigested or digested with N-glycanase (for 4h at 37^0^C) was loaded in each lane and fractionated using 7.5% SDS/PAGE. Each experiment shown here was performed at least three times for confirmation; data for each figure are from a single representative experiment.


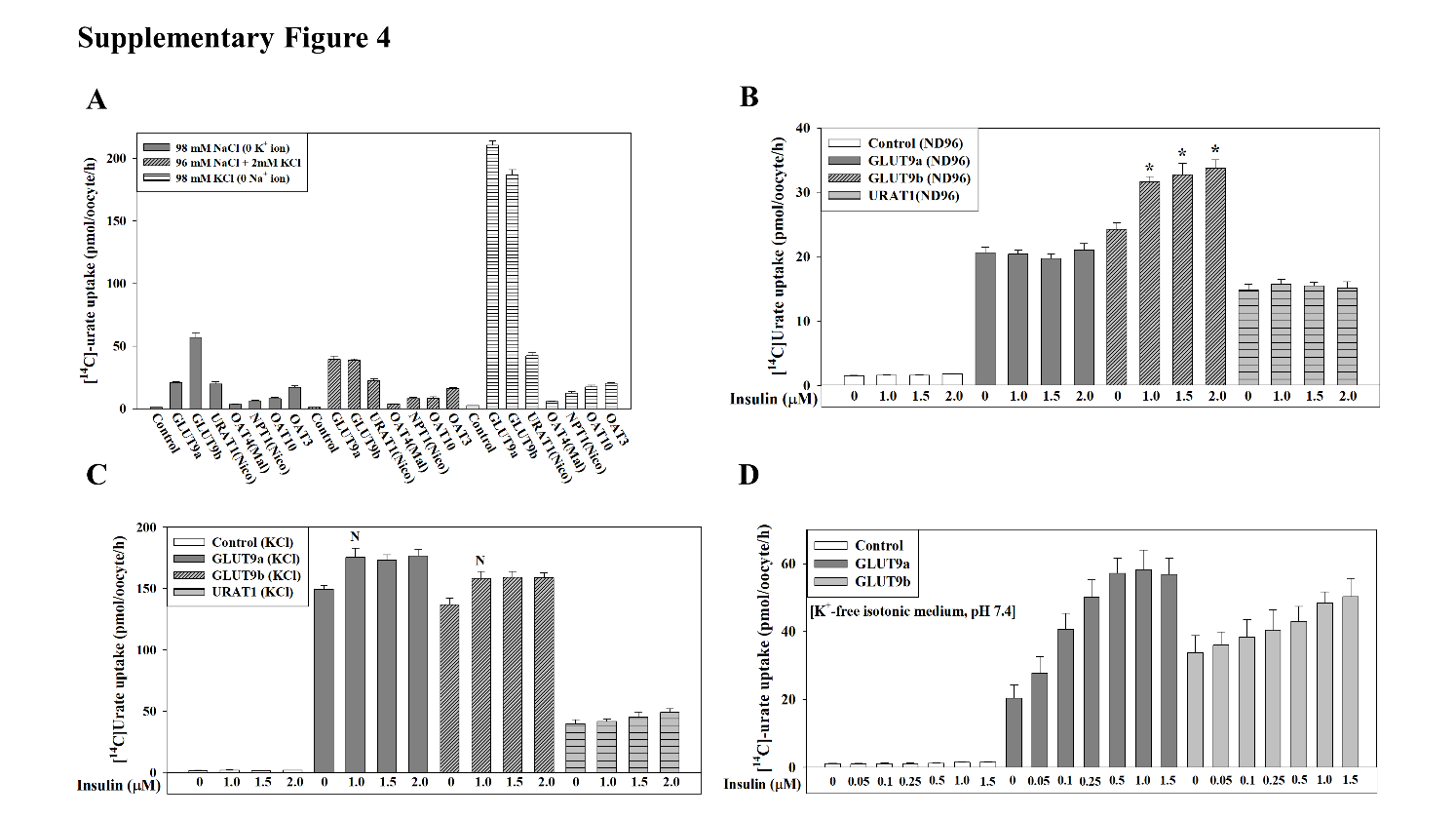


**Supplementary Figure 4**

Urate transport activities of human/mouse urate transporters (GLUT9a/b, URAT1, OAT4, NPT1, OAT10 and mOAT3) expressed in *Xenopus* *laevis* oocytes under three different experimental conditions and their response to extracellular insulin (1-2 µM). (**4A)** Urate transport activities of the indicated urate transporters were measured in K^+^-free isotonic medium (achieved by substituting 2 mM KCl by 2 mM NaCl in ND96 medium, pH 7.4), ND96 medium (see methods, pH 7.4) or Na^+^-free isotonic medium (achieved by substituting 96 mM NaCl by 96 mM KCl of ND96 medium, pH 7.4), containing 20 μM [^14^C]-urate, 1h after incubation at room temperature (~25^0^C) with orbital shaking. Each oocyte was microinjected with 50 nl of cRNA solution (~25ng of cRNA) of each indicated urate transporter and then incubated in ND96 medium supplemented with 2**.**5 mM pyruvate for 48 h at 16-18^o^C. Oocytes expressing URAT1, OAT10 or NPT1 were preloaded with nicotinate (Nico) and oocytes expressing OAT4 were preloaded with maleate (Mal) by microinjection of 50 nl of 100 mM nicotinate/maleate 2h before urate uptake. Prior to [^14^C]-urate uptake, oocytes were incubated in ND96 medium (pH 7.4), K^+^-free isotonic medium or in Na^+^-free isotonic medium for 30 min at room temperature (~25^0^C). (**4B** and **4C)** Urate transport activities of human urate transporters (GLUT9a/b, URAT1) were measured in ND96 medium (pH 7.4) (**4B**) or in Na^+^-free isotonic medium (**4C**) containing 20 μM [^14^C]-urate in the absence or presence of 1-2 µM extracellular recombinant human insulin. (**4D**) Dose-response plots of the effect of insulin on GLUT9: Urate uptake activities of GLUT9a and GLUT9b were measured in K^+^-free isotonic medium (pH 7.4) in presence of varying concentrations of insulin (0.05 -1.5 mM). Oocytes expressing URAT1 were preloaded with nicotinate (Nico) by microinjection of 50 nl of 100 mM nicotinate 2h before urate uptake. All data are mean $\pm$
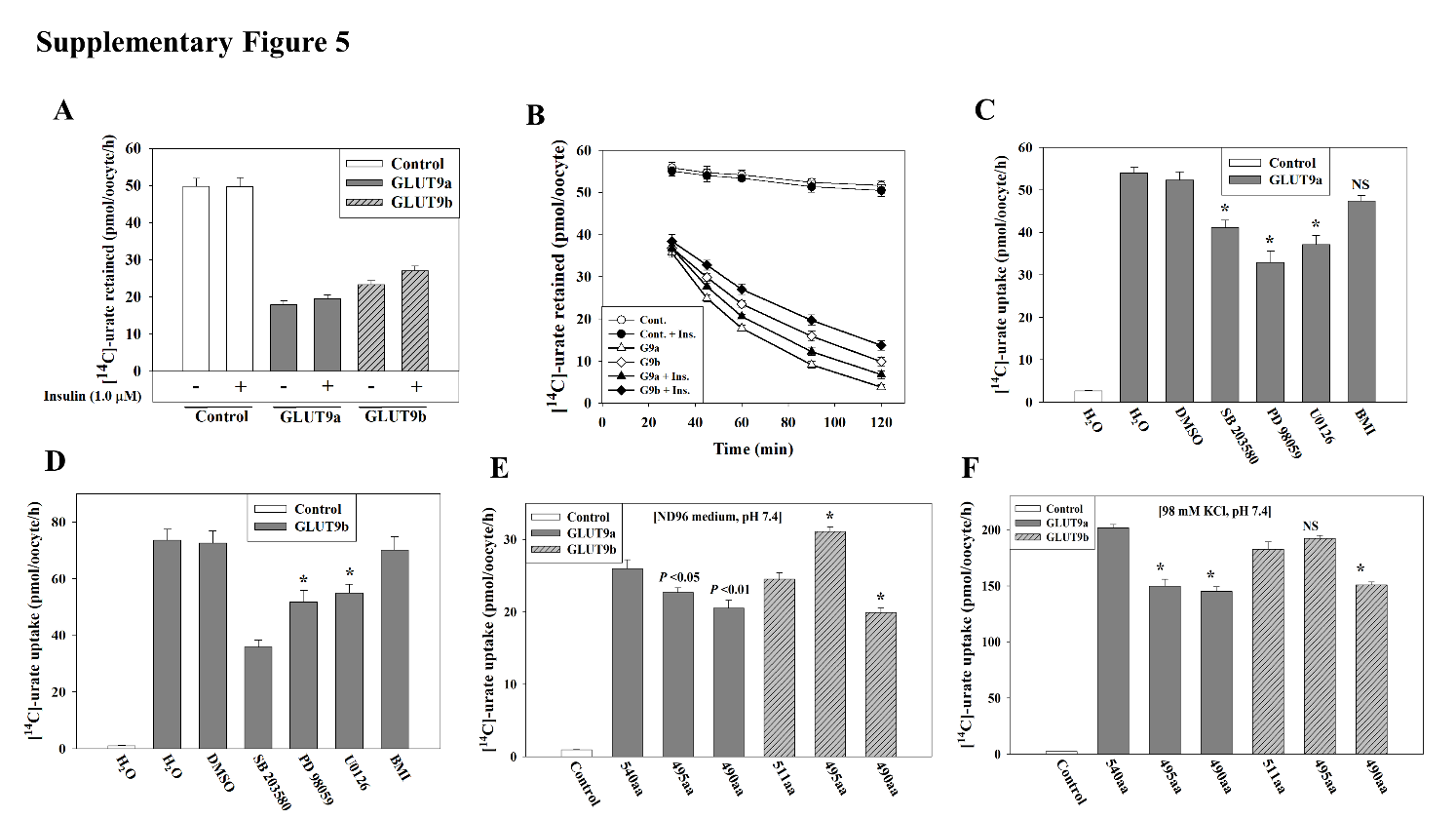
s.e.m. with n = 12–15 oocytes per group.

**Supplementary Figure 5**

Basal and insulin-activated urate transport activities of GLUT9 isoforms and N-terminal deletion mutants expressed in oocytes: **(5A** and **B)** The [^14^C]-urate efflux activities of GLUT9 isoforms, expressed in oocytes, in K^+^-free isotonic medium (pH 7.4; see methods) at ~25^0^C in the absence or presence of Insulin (1**.**0 µM) and the time course plot of its [^14^C]-urate efflux activities. (**5C** and **5D)** The basal urate transport activity of GLUT9a (**5C**) or GLUT9b (**5D**) expressed in oocytes, was measured 1h after incubation in the absence or presence of p38 MAPK inhibitor (SB 203580; 10 µM), MEK/ERK inhibitor (PD 98059/U0126; 20 µM) or protein kinase C inhibitor (bisindolylmaleimide I hydrochloride, or BMI; 10 µM) in the K^+^-free isotonic extracellular medium. (**5E** and **5F)** The basal urate transport activities of N-terminal deletion mutants of GLUT9a/b, expressed in oocytes, were measured in ND96 medium (**5E**) or in Na^+^-free isotonic medium (**5F**). The uptake of [^14^C]-urate was measured in K^+^-free/ND96/Na^+^-free isotonic medium containing 20 μM [^14^C]-urate after 1h of incubation at room temperature (~25^0^C) with orbital shaking. All data are mean $\pm$ s.e.m. with n = 12–15 oocytes per group.


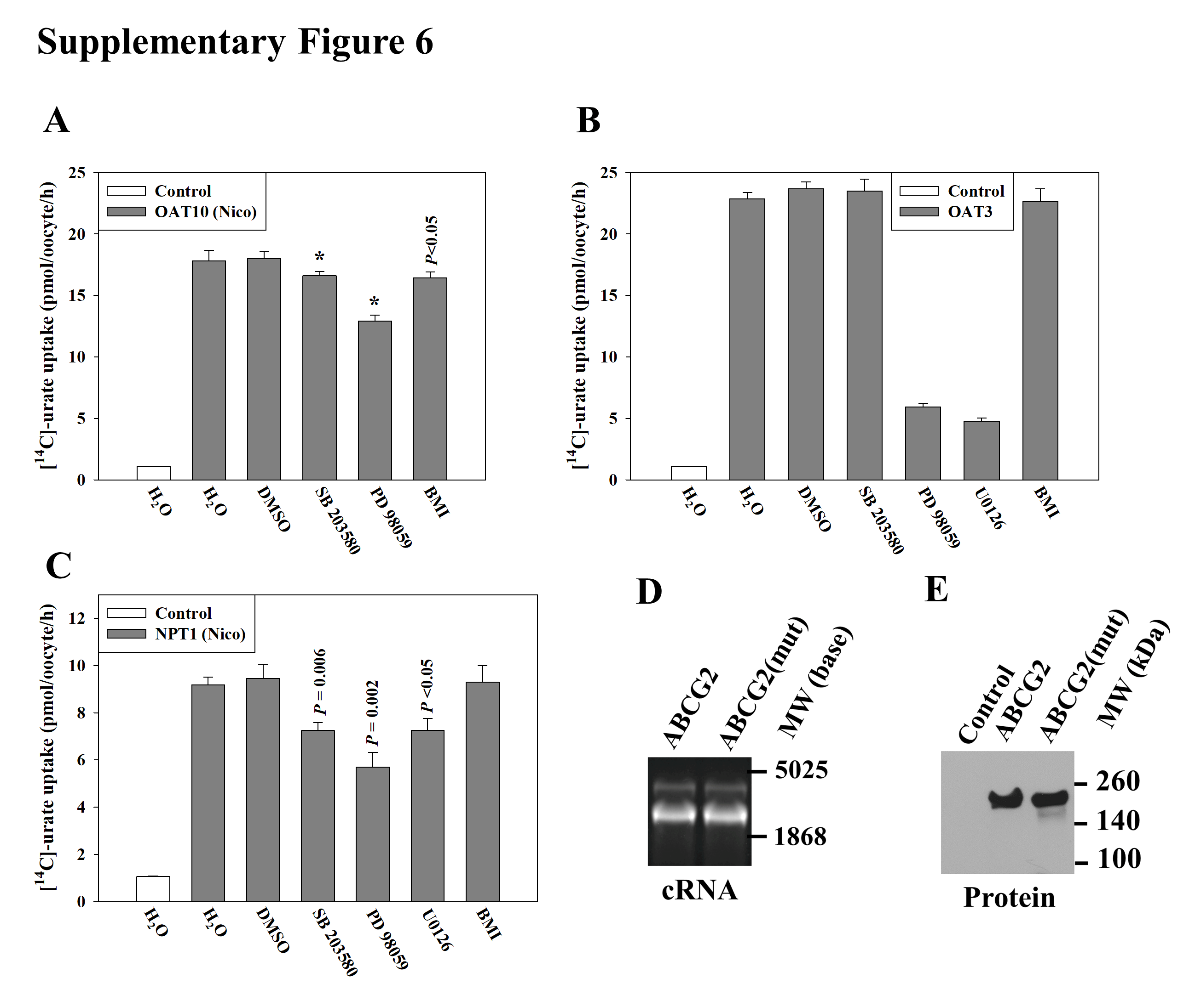


**Supplementary Figure 6**

Specific inhibitors of p38 MAPK and ERK pathways affect the basal urate transport activities of OAT10, OAT3 and NPT1 expressed in oocytes. (**6A** and **6B)** The basal urate transport activity of human OAT10 (**6A**), mouse OAT3 (**6B**) or human NPT1 (**6C**), expressed in oocytes, was measured in the absence and presence of p38 MAPK inhibitor (SB 203580; 10 µM), MEK/ERK inhibitor (PD 98059/U0126; 20 µM) or protein kinase C inhibitor (bisindolylmaleimide I hydrochloride, or BMI; 10 µM) in K^+^-free isotonic medium containing 20 μM [^14^C]-urate after 1h of incubation at room temperature (~25^0^C) with orbital shaking. Oocytes expressing OAT10 or NPT1 were preloaded with nicotinate (Nico) by microinjection of 50 nl of 100 mM nicotinate 2h before urate uptake. All data are mean $\pm$ s.e.m. with n = 12–15 oocytes per group. (**6D)** *In vitro* synthesized cRNAs of the urate transporter ABCG2 or its mutant (Q141K) were analyzed by 1.0% agarose-formaldehyde gel electrophoresis followed by ethidium bromide staining to assess their integrity before microinjection into each oocyte. (**6E)** Western blot analyses of the expression of ABCG2 or its mutant protein (Q141K), in *Xenopus* oocytes after microinjection of their respective cRNA and incubation for 48 hours in ND96 medium supplemented with pyruvate (see methods), using rabbit anti-ABCG2 antibody. About 20 µg of the total proteins from oocyte lysates was loaded in each lane and fractionated using 7.5% SDS/PAGE. Each experiment shown here was performed at least three times for confirmation; data for each figure are from a single representative experiment.


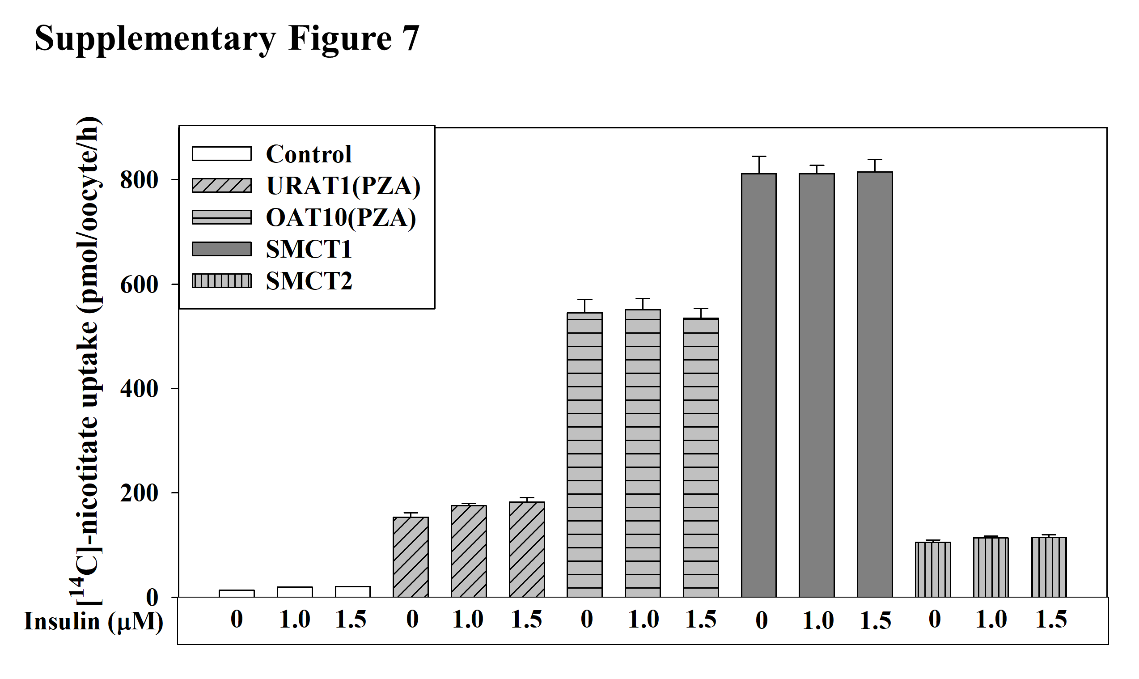


**Supplementary Figure 7**

Insulin has no effect on nicotinate uptake mediated by urate transporters (URAT1 and OAT10) or nicotinate transporters (SMCT1 and SMCT2). The [^14^C]-nicotinate uptake activity of human URAT1, OAT10, SMCT1 or SMCT2, expressed in oocytes, was measured in the absence and presence of insulin (1.0-1.5 µM) in K^+^-free isotonic medium containing 40 µM [^14^C]-nicotinate after 1h of incubation at room temperature (~25^0^C). Oocytes, expressing URAT1 or OAT10, were preloaded with pyrazinoate (PZA) by microinjection of 50 nl of 100 mM pyrazinoate 2h before urate uptake. Prior to the nicotinate uptake experiment, oocytes were pre-incubated for 30 min at ~25^0^C in a K^+^-free isotonic uptake medium (pH 7.4; see methods) without or with insulin (1.0 µM). All data are mean $\pm$ s.e.m. with n = 12–15 oocytes per group. Each experiment shown here was performed at least three times for confirmation; data for each figure are from a single representative experiment.


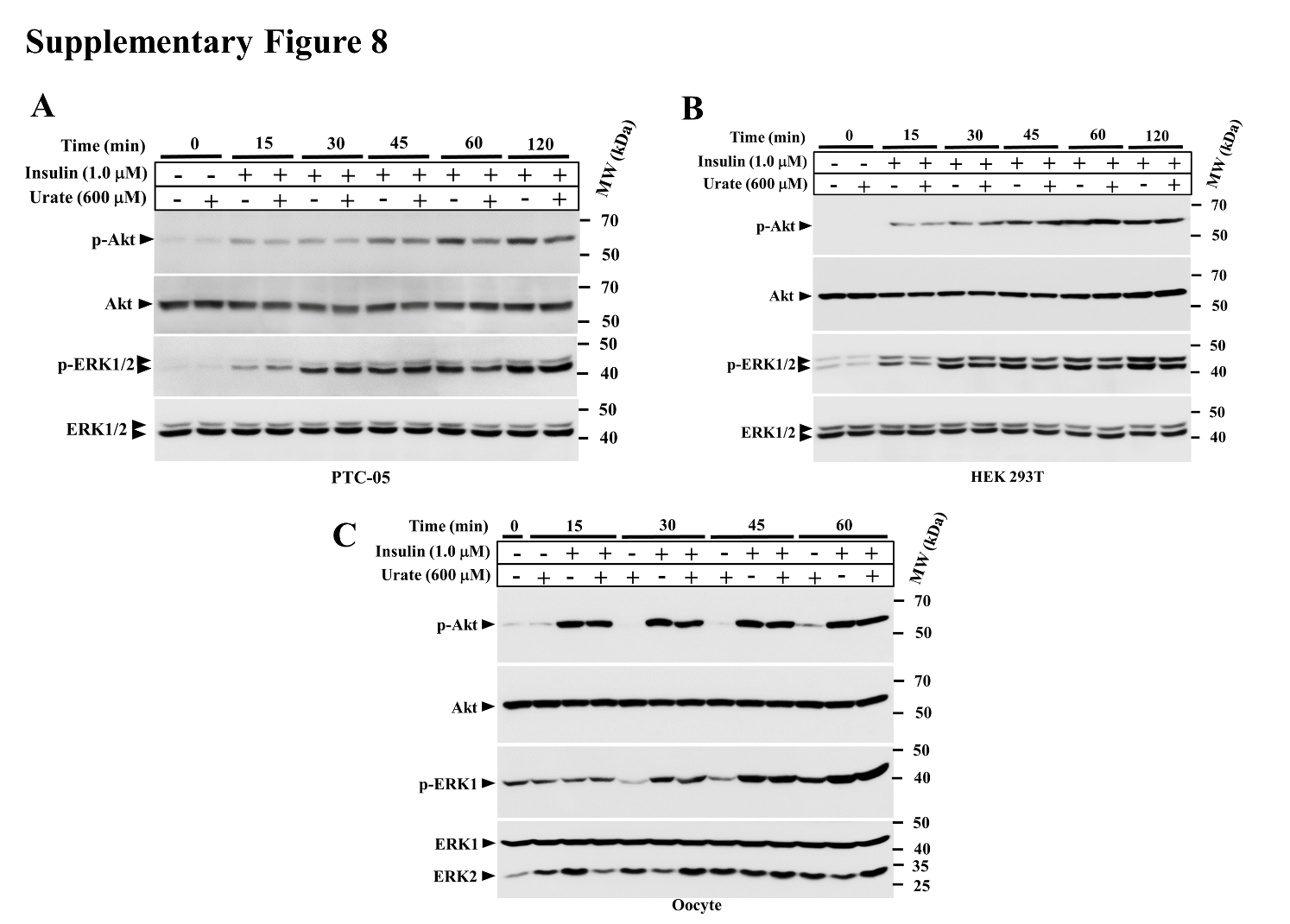


**Supplementary Figure 8**

Urate has no significant effect on insulin-activation of Akt and ERK: (**A** and **B**) Activation of Akt by insulin signaling is not inhibited in presence of urate (600 µM) in PTC-05 cells (**A**), HEK 293T cells (**B**) and oocytes (**C**). Western blot analyses of cell lysates (30 µg/lane) of PTC-05, HEK 293T cells or oocytes treated without or with insulin (1.0 µM) in K^+^-free and serum-free isotonic medium (see methods) for varying intervals of time at room temperature (~25^0^C) in the absence or presence of urate (600 µM) using rabbit anti-phospho-Akt (Ser473) antibody, anti-phospho-ERK1/2 (Thr202/Tyr204) antibody, anti-Akt antibody or anti-ERK1/2 antibody. Proteins were fractionated using 8.0% SDS/PAGE. Each experiment shown here was performed at least three times for confirmation; data for each figure are from a single representative experiment.
